# Supplementary material for: Structural basis for ryanodine receptor type 2 leak in heart failure and arrhythmogenic disorders
Source: Nat Commun. 2024 Sep 15;15:8080. doi: 10.1038/s41467-024-51791-y (PMC11402997; doi:10.1038/s41467-024-51791-y)
Supplement: Supplementary file 1 — Supplementary Information [file 41467_2024_51791_MOESM1_ESM.pdf]

Supplementary information

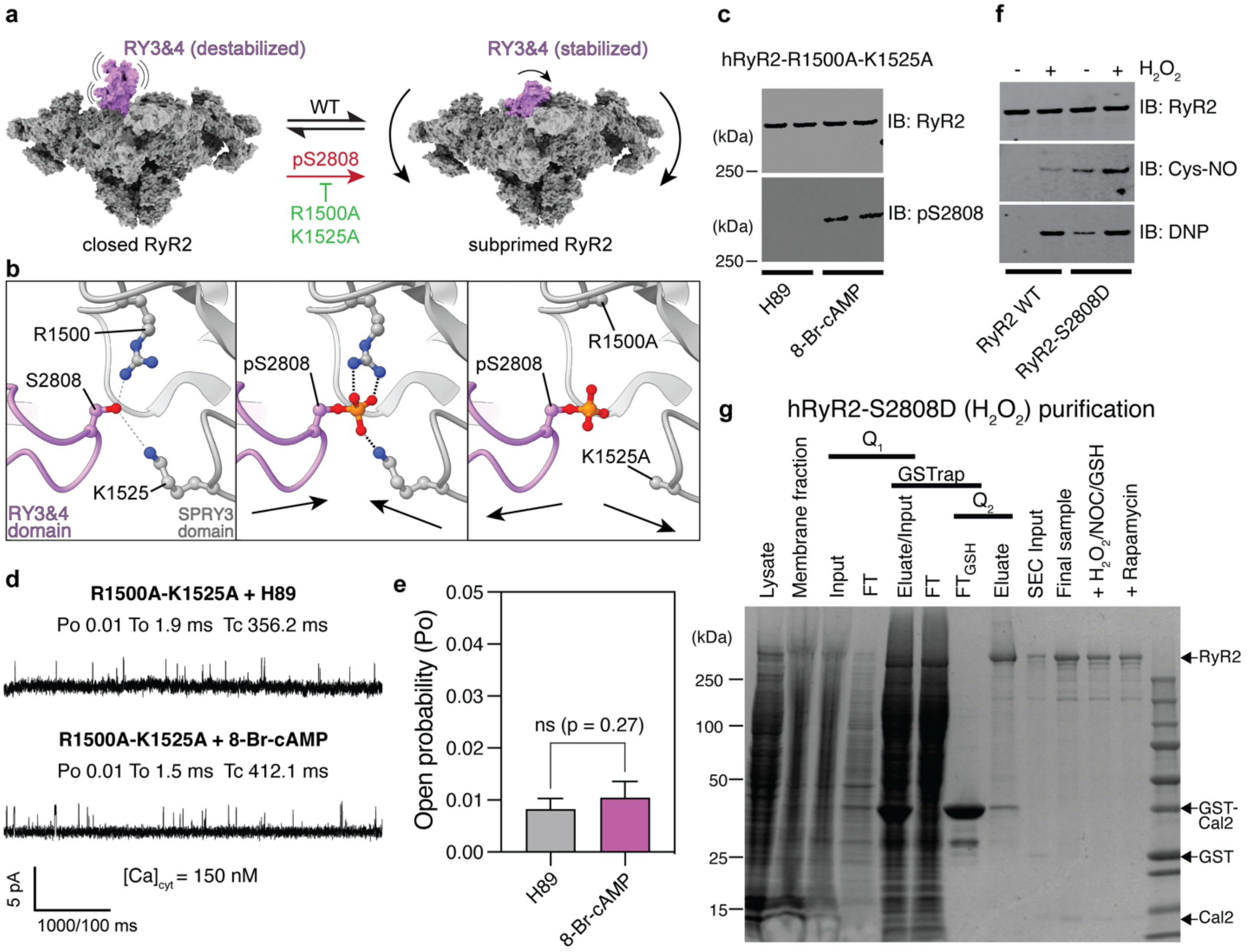

Supplementary Fig. 1. Phosphorylation of residue RyR2-S2808 induces the subprimed state and post-translational modifications. a Schematic equilibrium of the different conformations of RyR2. Panel adapted from Marco C. Miotto et al., Structural analyses of human ryanodine recepor type 2 channels reveal the mechanisms for sudden cardiac death and treatment. Sci. Adv. 8, eabo1272(2022).DOI:10.1126/sciadv.abo1272 under a CC BY license: https://creativecommons.org/licenses/by/4.0/.[10] The conformation of the destabilized RY3&4 domain (phosphorylation domain) was modelled with Coot to illustrate our hypothesis. **b** Close-up view of the phosphorylation loop (residues 2803-2819 modelled with Coot) of the WT RyR2 (left), PKA-phosphorylated RyR2 (middle), and RyR2-R1500A-K1525A (right). The dashed lines represent the hydrogen and ionic bonds, and the arrows represent the expected domain movements. **c** Immunoblots of microsomes prepared from HEK293 expressing RyR2-R1500A-K1525A treated with either 30 µM H89 overnight or 100 µM 8-Br-cAMP for 30 min, showing phosphorylation of residue S2808. **d,e** PKA phosphorylation fails to increase the open probability (Po) of single RyR2-R1500A-K1525A channels. Single-channel current recordings traces from recombinant RyR2-R1500A-K1525A at Ca^{2+} 150 nM, with H89 or 8-Br-cAMP treatment.

Opening events are shown as upward deflections. Po: opening probability, To : meantime open, Tc : meantime closed. **f** Immunoblots of microsomes prepared from HEK293 expressing WT RyR2 or RyR2-S2808D treated with 1 mM H_2O_2 for 30 min, showing oxidation and nitrosylation of RyR2. **g** SDS-PAGE of human recombinant RyR2-S2808D purified from HEK293 cells treated with 1 mM H_2O_2 for 30 min.

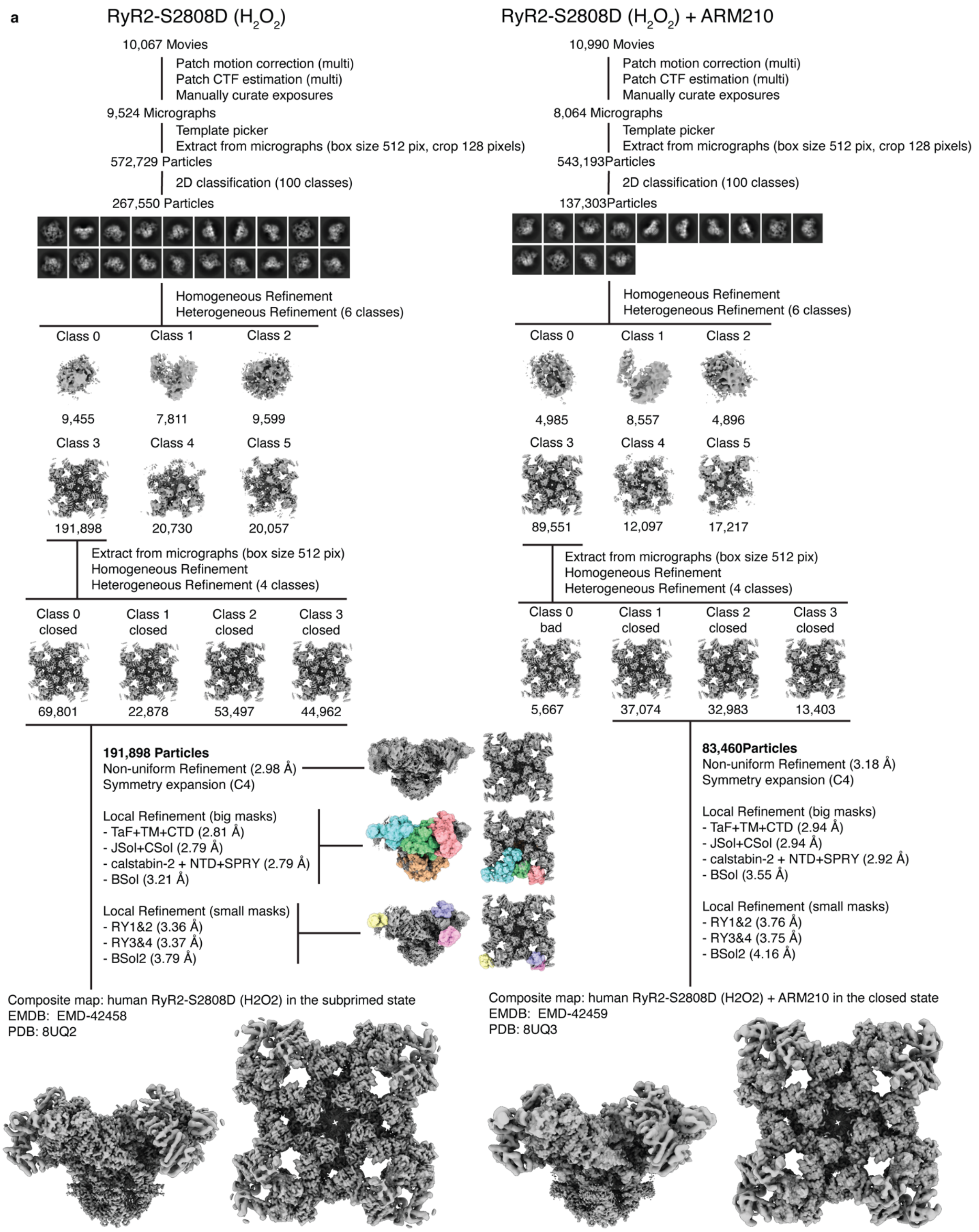
a
RyR2-S2808D (H_2O_2)
10,067 Movies
Patch motion correction (multi)
Patch CTF estimation (multi)
Manually curate exposures
9,524 Micrographs
Template picker
Extract from micrographs (box size 512 pix, crop 128 pixels)
572,729 Particles
2D classification (100 classes)
267,550 Particles
Homogeneous Refinement
Heterogeneous Refinement (6 classes)
Class 0
Class 1
Class 2
9,455
7,811
9,599
Class 3
Class 4
Class 5
191,898
20,730
20,057
Extract from micrographs (box size 512 pix)
Homogeneous Refinement
Heterogeneous Refinement (4 classes)
Class 0 closed
Class 1 closed
Class 2 closed
Class 3 closed
69,801
22,878
53,497
44,962
191,898 Particles
Non-uniform Refinement (2.98 Å)
Symmetry expansion (C4)
Local Refinement (big masks)
- TaF+TM+CTD (2.81 Å)
- JSol+CSol (2.79 Å)
- calstabin-2 + NTD+SPRY (2.79 Å)
- BSol (3.21 Å)
Local Refinement (small masks)
- RY1&2 (3.36 Å)
- RY3&4 (3.37 Å)
- BSol2 (3.79 Å)
Composite map: human RyR2-S2808D (H2O2) in the subprimed state
EMDB: EMD-42458
PDB: 8UQ2
RyR2-S2808D (H_2O_2) + ARM210
10,990 Movies
Patch motion correction (multi)
Patch CTF estimation (multi)
Manually curate exposures
8,064 Micrographs
Template picker
Extract from micrographs (box size 512 pix, crop 128 pixels)
543,193Particles
2D classification (100 classes)
137,303Particles
Homogeneous Refinement
Heterogeneous Refinement (6 classes)
Class 0
Class 1
Class 2
4,985
8,557
4,896
Class 3
Class 4
Class 5
89,551
12,097
17,217
Extract from micrographs (box size 512 pix)
Homogeneous Refinement
Heterogeneous Refinement (4 classes)
Class 0 bad
Class 1 closed
Class 2 closed
Class 3 closed
5,667
37,074
32,983
13,403
83,460Particles
Non-uniform Refinement (3.18 Å)
Symmetry expansion (C4)
Local Refinement (big masks)
- TaF+TM+CTD (2.94 Å)
- JSol+CSol (2.94 Å)
- calstabin-2 + NTD+SPRY (2.92 Å)
- BSol (3.55 Å)
Local Refinement (small masks)
- RY1&2 (3.76 Å)
- RY3&4 (3.75 Å)
- BSol2 (4.16 Å)
Composite map: human RyR2-S2808D (H2O2) + ARM210 in the closed state
EMDB: EMD-42459
PDB: 8UQ3

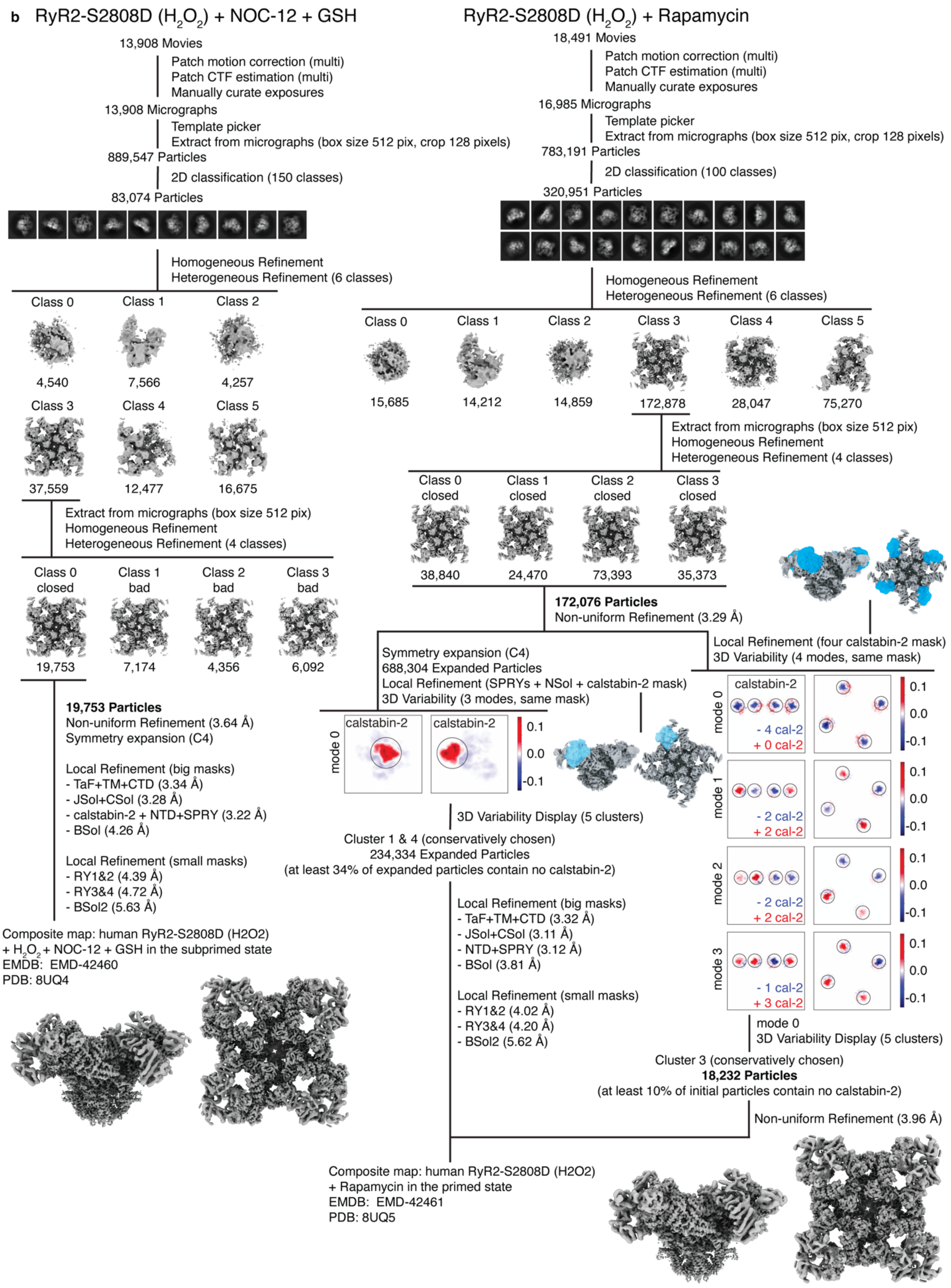
b
RyR2-S2808D (H_2O_2) + NOC-12 + GSH
13,908 Movies
Patch motion correction (multi)
Patch CTF estimation (multi)
Manually curate exposures
13,908 Micrographs
Template picker
Extract from micrographs (box size 512 pix, crop 128 pixels)
889,547 Particles
2D classification (150 classes)
83,074 Particles
Homogeneous Refinement
Heterogeneous Refinement (6 classes)
Class 0
Class 1
Class 2
4,540
7,566
4,257
Class 3
Class 4
Class 5
37,559
12,477
16,675
Extract from micrographs (box size 512 pix)
Homogeneous Refinement
Heterogeneous Refinement (4 classes)
Class 0 closed
Class 1 bad
Class 2 bad
Class 3 bad
19,753
7,174
4,356
6,092
19,753 Particles
Non-uniform Refinement (3.64 Å)
Symmetry expansion (C4)
Local Refinement (big masks)
- TaF+TM+CTD (3.34 Å)
- JSol+CSol (3.28 Å)
- calstabin-2 + NTD+SPRY (3.22 Å)
- BSol (4.26 Å)
Local Refinement (small masks)
- RY1&2 (4.39 Å)
- RY3&4 (4.72 Å)
- BSol2 (5.63 Å)
Composite map: human RyR2-S2808D (H2O2) + H_2O_2 + NOC-12 + GSH in the subprimed state
EMDB: EMD-42460
PDB: 8UQ4
RyR2-S2808D (H_2O_2) + Rapamycin
18,491 Movies
Patch motion correction (multi)
Patch CTF estimation (multi)
Manually curate exposures
16,985 Micrographs
Template picker
Extract from micrographs (box size 512 pix, crop 128 pixels)
783,191 Particles
2D classification (100 classes)
320,951 Particles
Homogeneous Refinement
Heterogeneous Refinement (6 classes)
Class 0
Class 1
Class 2
Class 3
Class 4
Class 5
15,685
14,212
14,859
172,878
28,047
75,270
Extract from micrographs (box size 512 pix)
Homogeneous Refinement
Heterogeneous Refinement (4 classes)
Class 0 closed
Class 1 closed
Class 2 closed
Class 3 closed
38,840
24,470
73,393
35,373
172,076 Particles
Non-uniform Refinement (3.29 Å)
Local Refinement (four calstabin-2 mask)
3D Variability (4 modes, same mask)
Symmetry expansion (C4)
688,304 Expanded Particles
Local Refinement (SPRYs + NSol + calstabin-2 mask)
3D Variability (3 modes, same mask)
mode 0
calstabin-2
calstabin-2
0.1
0.0
-0.1
3D Variability Display (5 clusters)
Cluster 1 & 4 (conservatively chosen)
234,334 Expanded Particles
(at least 34% of expanded particles contain no calstabin-2)
Local Refinement (big masks)
- TaF+TM+CTD (3.32 Å)
- JSol+CSol (3.11 Å)
- NTD+SPRY (3.12 Å)
- BSol (3.81 Å)
Local Refinement (small masks)
- RY1&2 (4.02 Å)
- RY3&4 (4.20 Å)
- BSol2 (5.62 Å)
mode 0
- 4 cal-2
+ 0 cal-2
mode 1
- 2 cal-2
+ 2 cal-2
mode 2
- 2 cal-2
+ 2 cal-2
mode 3
- 1 cal-2
+ 3 cal-2
mode 0
3D Variability Display (5 clusters)
Cluster 3 (conservatively chosen)
18,232 Particles
(at least 10% of initial particles contain no calstabin-2)
Non-uniform Refinement (3.96 Å)
Composite map: human RyR2-S2808D (H2O2) + Rapamycin in the primed state
EMDB: EMD-42461
PDB: 8UQ5

c

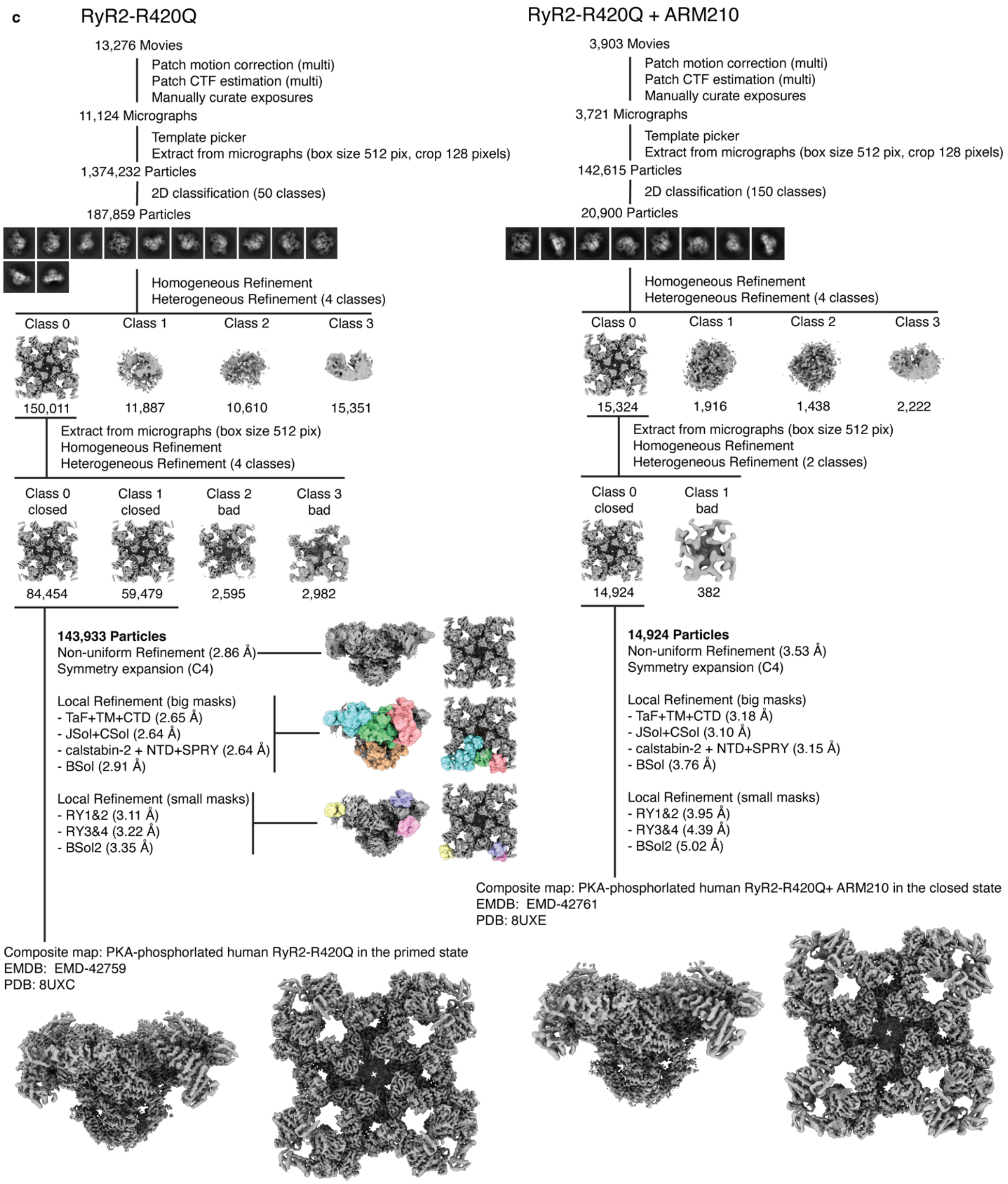

RyR2-R420Q
13,276 Movies
Patch motion correction (multi)
Patch CTF estimation (multi)
Manually curate exposures
11,124 Micrographs
Template picker
Extract from micrographs (box size 512 pix, crop 128 pixels)
1,374,232 Particles
2D classification (50 classes)
187,859 Particles
Homogeneous Refinement
Heterogeneous Refinement (4 classes)
Class 0
Class 1
Class 2
Class 3
150,011
11,887
10,610
15,351
Extract from micrographs (box size 512 pix)
Homogeneous Refinement
Heterogeneous Refinement (4 classes)
Class 0 closed
Class 1 closed
Class 2 bad
Class 3 bad
84,454
59,479
2,595
2,982
143,933 Particles
Non-uniform Refinement (2.86 Å)
Symmetry expansion (C4)
Local Refinement (big masks)
- TaF+TM+CTD (2.65 Å)
- JSol+CSol (2.64 Å)
- calstabin-2 + NTD+SPRY (2.64 Å)
- BSol (2.91 Å)
Local Refinement (small masks)
- RY1&2 (3.11 Å)
- RY3&4 (3.22 Å)
- BSol2 (3.35 Å)
Composite map: PKA-phosphorlated human RyR2-R420Q in the primed state
EMDB: EMD-42759
PDB: 8UXC
RyR2-R420Q + ARM210
3,903 Movies
Patch motion correction (multi)
Patch CTF estimation (multi)
Manually curate exposures
3,721 Micrographs
Template picker
Extract from micrographs (box size 512 pix, crop 128 pixels)
142,615 Particles
2D classification (150 classes)
20,900 Particles
Homogeneous Refinement
Heterogeneous Refinement (4 classes)
Class 0
Class 1
Class 2
Class 3
15,324
1,916
1,438
2,222
Extract from micrographs (box size 512 pix)
Homogeneous Refinement
Heterogeneous Refinement (2 classes)
Class 0 closed
Class 1 bad
14,924
382
14,924 Particles
Non-uniform Refinement (3.53 Å)
Symmetry expansion (C4)
Local Refinement (big masks)
- TaF+TM+CTD (3.18 Å)
- JSol+CSol (3.10 Å)
- calstabin-2 + NTD+SPRY (3.15 Å)
- BSol (3.76 Å)
Local Refinement (small masks)
- RY1&2 (3.95 Å)
- RY3&4 (4.39 Å)
- BSol2 (5.02 Å)
Composite map: PKA-phosphorlated human RyR2-R420Q+ ARM210 in the closed state
EMDB: EMD-42761
PDB: 8UXE

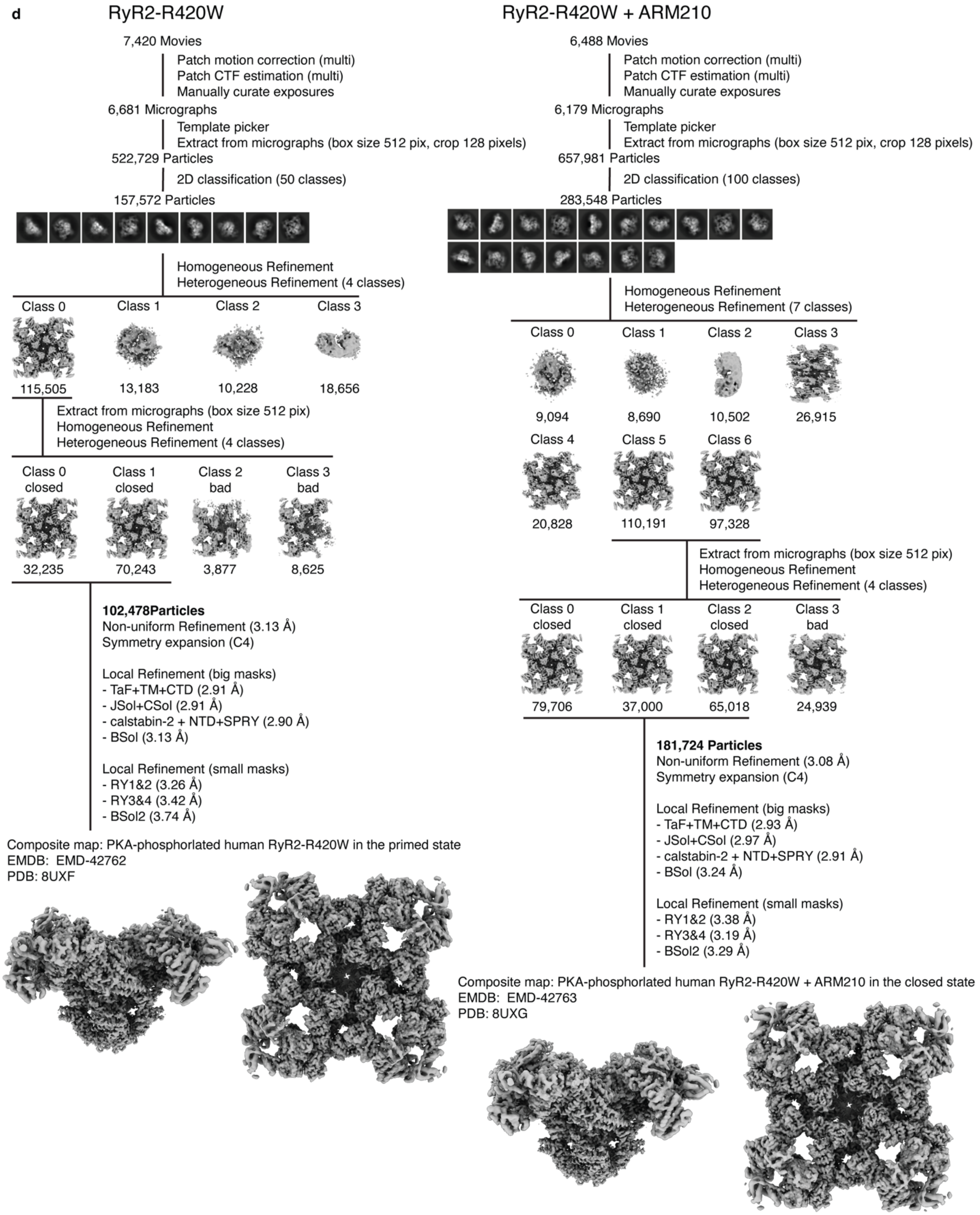
d
RyR2-R420W
7,420 Movies
Patch motion correction (multi)
Patch CTF estimation (multi)
Manually curate exposures
6,681 Micrographs
Template picker
Extract from micrographs (box size 512 pix, crop 128 pixels)
522,729 Particles
2D classification (50 classes)
157,572 Particles
Homogeneous Refinement
Heterogeneous Refinement (4 classes)
Class 0
Class 1
Class 2
Class 3
115,505
13,183
10,228
18,656
Extract from micrographs (box size 512 pix)
Homogeneous Refinement
Heterogeneous Refinement (4 classes)
Class 0 closed
Class 1 closed
Class 2 bad
Class 3 bad
32,235
70,243
3,877
8,625
102,478Particles
Non-uniform Refinement (3.13 Å)
Symmetry expansion (C4)
Local Refinement (big masks)
- TaF+TM+CTD (2.91 Å)
- JSol+CSol (2.91 Å)
- calstabin-2 + NTD+SPRY (2.90 Å)
- BSol (3.13 Å)
Local Refinement (small masks)
- RY1&2 (3.26 Å)
- RY3&4 (3.42 Å)
- BSol2 (3.74 Å)
Composite map: PKA-phosphorlated human RyR2-R420W in the primed state
EMDB: EMD-42762
PDB: 8UXF
RyR2-R420W + ARM210
6,488 Movies
Patch motion correction (multi)
Patch CTF estimation (multi)
Manually curate exposures
6,179 Micrographs
Template picker
Extract from micrographs (box size 512 pix, crop 128 pixels)
657,981 Particles
2D classification (100 classes)
283,548 Particles
Homogeneous Refinement
Heterogeneous Refinement (7 classes)
Class 0
Class 1
Class 2
Class 3
9,094
8,690
10,502
26,915
Class 4
Class 5
Class 6
20,828
110,191
97,328
Extract from micrographs (box size 512 pix)
Homogeneous Refinement
Heterogeneous Refinement (4 classes)
Class 0 closed
Class 1 closed
Class 2 closed
Class 3 bad
79,706
37,000
65,018
24,939
181,724 Particles
Non-uniform Refinement (3.08 Å)
Symmetry expansion (C4)
Local Refinement (big masks)
- TaF+TM+CTD (2.93 Å)
- JSol+CSol (2.97 Å)
- calstabin-2 + NTD+SPRY (2.91 Å)
- BSol (3.24 Å)
Local Refinement (small masks)
- RY1&2 (3.38 Å)
- RY3&4 (3.19 Å)
- BSol2 (3.29 Å)
Composite map: PKA-phosphorlated human RyR2-R420W + ARM210 in the closed state
EMDB: EMD-42763
PDB: 8UXG

e

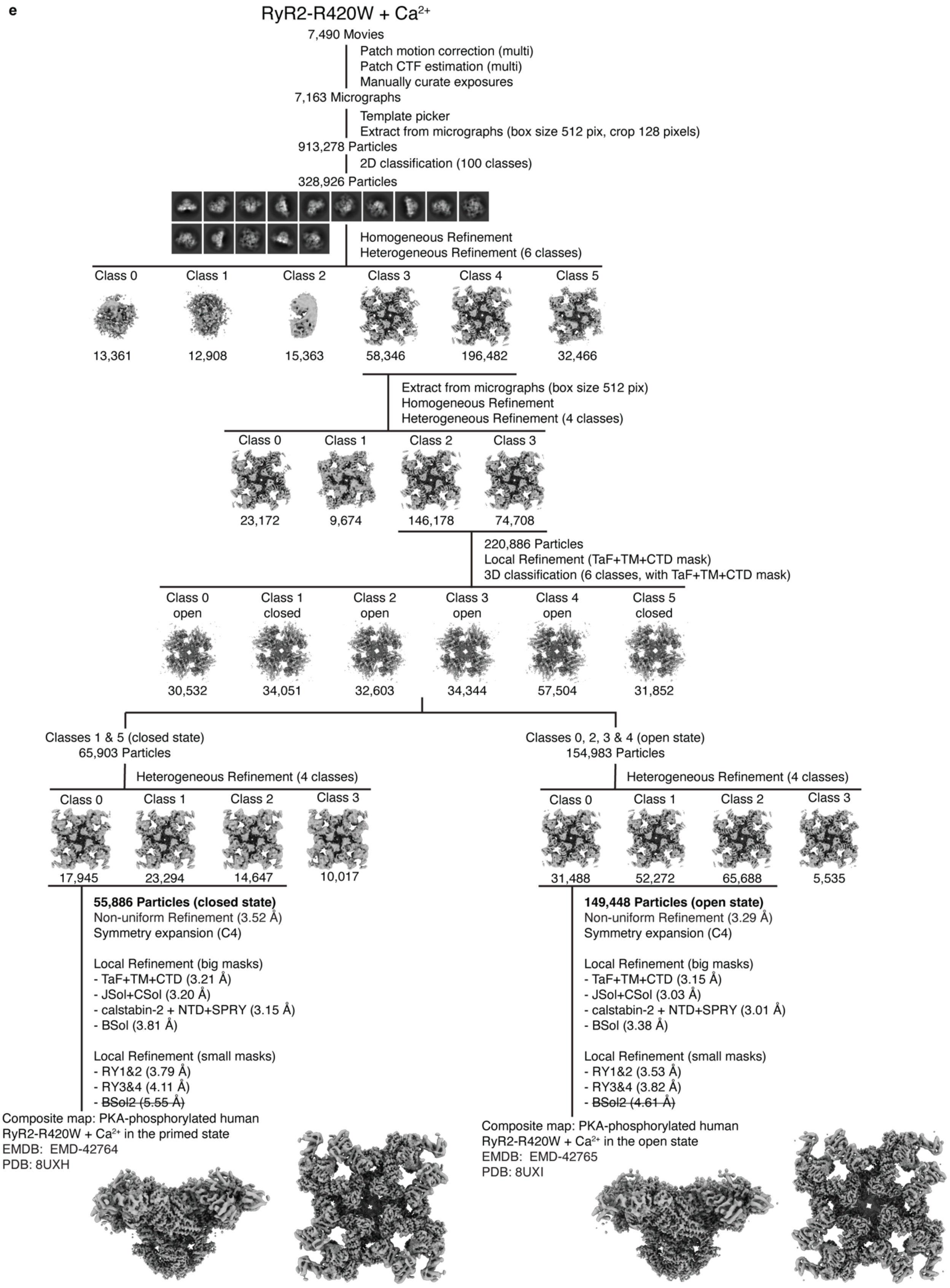
RyR2-R420W + Ca2+
7,490 Movies
Patch motion correction (multi)
Patch CTF estimation (multi)
Manually curate exposures
7,163 Micrographs
Template picker
Extract from micrographs (box size 512 pix, crop 128 pixels)
913,278 Particles
2D classification (100 classes)
328,926 Particles
Homogeneous Refinement
Heterogeneous Refinement (6 classes)
Class 0
Class 1
Class 2
Class 3
Class 4
Class 5
13,361
12,908
15,363
58,346
196,482
32,466
Extract from micrographs (box size 512 pix)
Homogeneous Refinement
Heterogeneous Refinement (4 classes)
Class 0
Class 1
Class 2
Class 3
23,172
9,674
146,178
74,708
220,886 Particles
Local Refinement (TaF+TM+CTD mask)
3D classification (6 classes, with TaF+TM+CTD mask)
Class 0 open
Class 1 closed
Class 2 open
Class 3 open
Class 4 open
Class 5 closed
30,532
34,051
32,603
34,344
57,504
31,852
Classes 1 & 5 (closed state)
65,903 Particles
Heterogeneous Refinement (4 classes)
Class 0
Class 1
Class 2
Class 3
17,945
23,294
14,647
10,017
55,886 Particles (closed state)
Non-uniform Refinement (3.52 Å)
Symmetry expansion (C4)
Local Refinement (big masks)
- TaF+TM+CTD (3.21 Å)
- JSol+CSol (3.20 Å)
- calstabin-2 + NTD+SPRY (3.15 Å)
- BSol (3.81 Å)
Local Refinement (small masks)
- RY1&2 (3.79 Å)
- RY3&4 (4.11 Å)
- ~~BSol2 (5.55 Å)~~
Composite map: PKA-phosphorylated human
RyR2-R420W + Ca2+ in the primed state
EMDB: EMD-42764
PDB: 8UXH
Classes 0, 2, 3 & 4 (open state)
154,983 Particles
Heterogeneous Refinement (4 classes)
Class 0
Class 1
Class 2
Class 3
31,488
52,272
65,688
5,535
149,448 Particles (open state)
Non-uniform Refinement (3.29 Å)
Symmetry expansion (C4)
Local Refinement (big masks)
- TaF+TM+CTD (3.15 Å)
- JSol+CSol (3.03 Å)
- calstabin-2 + NTD+SPRY (3.01 Å)
- BSol (3.38 Å)
Local Refinement (small masks)
- RY1&2 (3.53 Å)
- RY3&4 (3.82 Å)
- ~~BSol2 (4.61 Å)~~
Composite map: PKA-phosphorylated human
RyR2-R420W + Ca2+ in the open state
EMDB: EMD-42765
PDB: 8UXI

f

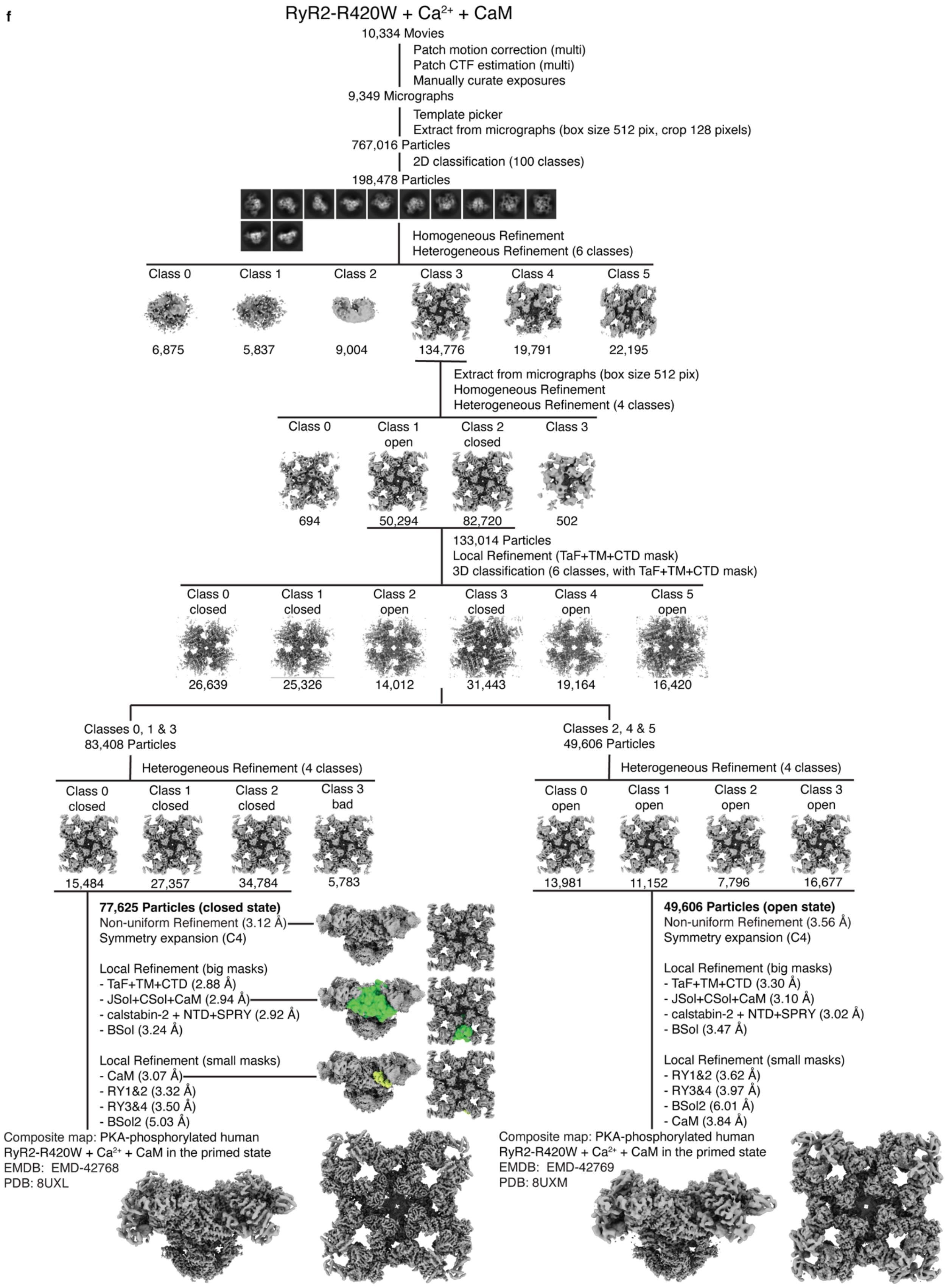
RyR2-R420W + Ca2+ + CaM
10,334 Movies
Patch motion correction (multi)
Patch CTF estimation (multi)
Manually curate exposures
9,349 Micrographs
Template picker
Extract from micrographs (box size 512 pix, crop 128 pixels)
767,016 Particles
2D classification (100 classes)
198,478 Particles
Homogeneous Refinement
Heterogeneous Refinement (6 classes)
Class 0 6,875
Class 1 5,837
Class 2 9,004
Class 3 134,776
Class 4 19,791
Class 5 22,195
Extract from micrographs (box size 512 pix)
Homogeneous Refinement
Heterogeneous Refinement (4 classes)
Class 0 694
Class 1 open 50,294
Class 2 closed 82,720
Class 3 502
133,014 Particles
Local Refinement (TaF+TM+CTD mask)
3D classification (6 classes, with TaF+TM+CTD mask)
Class 0 closed 26,639
Class 1 closed 25,326
Class 2 open 14,012
Class 3 closed 31,443
Class 4 open 19,164
Class 5 open 16,420
Classes 0, 1 & 3
83,408 Particles
Heterogeneous Refinement (4 classes)
Class 0 closed 15,484
Class 1 closed 27,357
Class 2 closed 34,784
Class 3 bad 5,783
77,625 Particles (closed state)
Non-uniform Refinement (3.12 Å)
Symmetry expansion (C4)
Local Refinement (big masks)
- TaF+TM+CTD (2.88 Å)
- JSol+CSol+CaM (2.94 Å)
- calstabin-2 + NTD+SPRY (2.92 Å)
- BSol (3.24 Å)
Local Refinement (small masks)
- CaM (3.07 Å)
- RY1&2 (3.32 Å)
- RY3&4 (3.50 Å)
- BSol2 (5.03 Å)
Composite map: PKA-phosphorylated human
RyR2-R420W + Ca2+ + CaM in the primed state
EMDB: EMD-42768
PDB: 8UXL
Classes 2, 4 & 5
49,606 Particles
Heterogeneous Refinement (4 classes)
Class 0 open 13,981
Class 1 open 11,152
Class 2 open 7,796
Class 3 open 16,677
49,606 Particles (open state)
Non-uniform Refinement (3.56 Å)
Symmetry expansion (C4)
Local Refinement (big masks)
- TaF+TM+CTD (3.30 Å)
- JSol+CSol+CaM (3.10 Å)
- calstabin-2 + NTD+SPRY (3.02 Å)
- BSol (3.47 Å)
Local Refinement (small masks)
- RY1&2 (3.62 Å)
- RY3&4 (3.97 Å)
- BSol2 (6.01 Å)
- CaM (3.84 Å)
Composite map: PKA-phosphorylated human
RyR2-R420W + Ca2+ + CaM in the primed state
EMDB: EMD-42769
PDB: 8UXM

Supplementary Fig. 2. Cryo-EM data processing. a-f Entire cryoSPARC processing of the cryo-EM datasets to obtain final composite maps of RyR2-S2808D, RyR2-R420Q, and RyR2-R420W variants in the different conditions and states. The number of particles and the resolution achieved for each refinement are shown for each step. The masks used are shown only in **a** but are identical in all processing procedures, except those including calstabin-2 (**b**) and CaM (**f**).

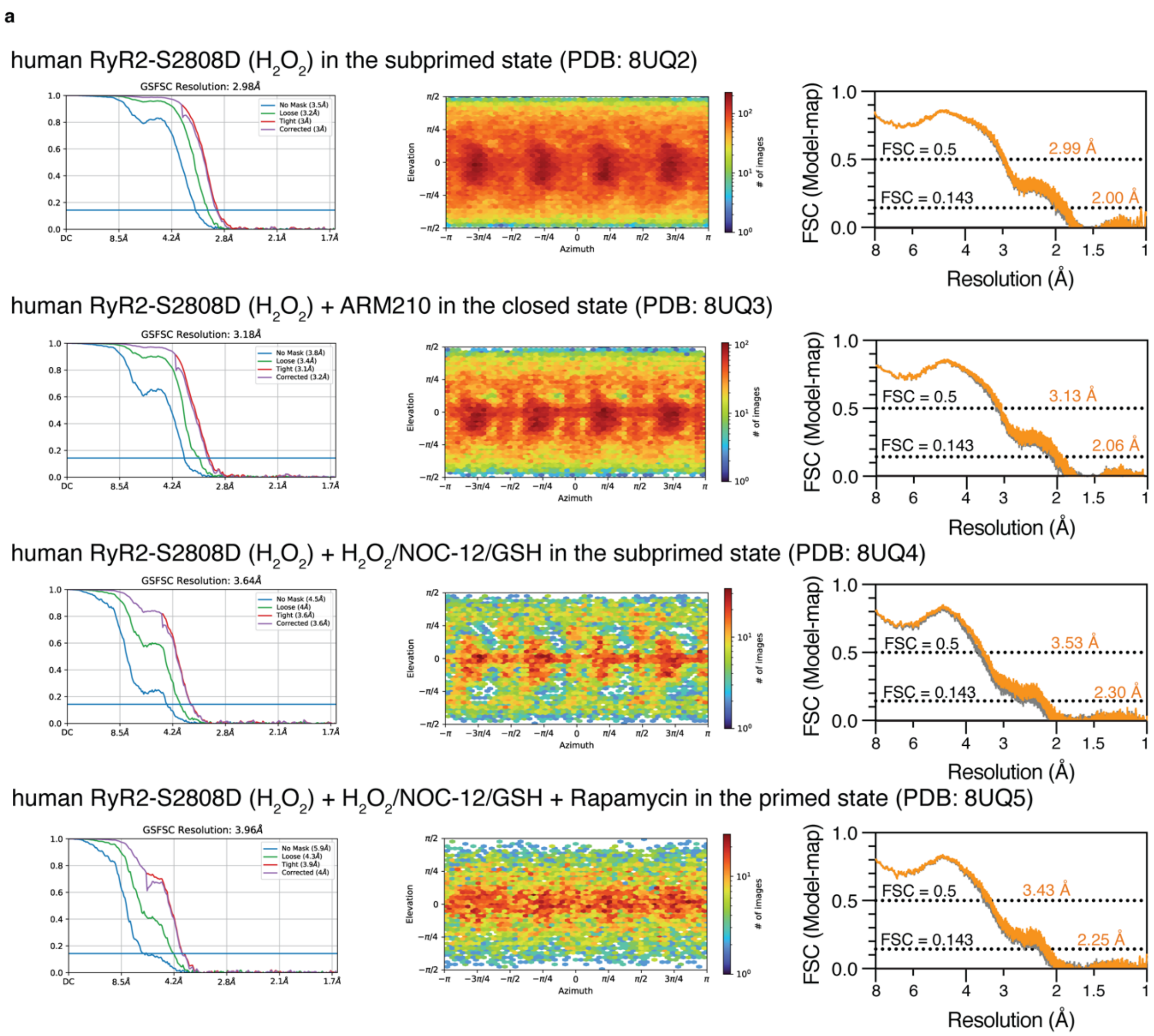
a
human RyR2-S2808D (H_2O_2) in the subprimed state (PDB: 8UQ2)
GSFSC Resolution: 2.98Å
No Mask (3.5Å)
Loose (3.2Å)
Tight (3Å)
Corrected (3Å)
Elevation
Azimuth
# of images
FSC (Model-map)
FSC = 0.5
2.99 Å
FSC = 0.143
2.00 Å
Resolution (Å)
human RyR2-S2808D (H_2O_2) + ARM210 in the closed state (PDB: 8UQ3)
GSFSC Resolution: 3.18Å
No Mask (3.8Å)
Loose (3.4Å)
Tight (3.1Å)
Corrected (3.2Å)
Elevation
Azimuth
# of images
FSC (Model-map)
FSC = 0.5
3.13 Å
FSC = 0.143
2.06 Å
Resolution (Å)
human RyR2-S2808D (H_2O_2) + H_2O_2/NOC-12/GSH in the subprimed state (PDB: 8UQ4)
GSFSC Resolution: 3.64Å
No Mask (4.5Å)
Loose (4Å)
Tight (3.6Å)
Corrected (3.6Å)
Elevation
Azimuth
# of images
FSC (Model-map)
FSC = 0.5
3.53 Å
FSC = 0.143
2.30 Å
Resolution (Å)
human RyR2-S2808D (H_2O_2) + H_2O_2/NOC-12/GSH + Rapamycin in the primed state (PDB: 8UQ5)
GSFSC Resolution: 3.96Å
No Mask (5.9Å)
Loose (4.3Å)
Tight (3.9Å)
Corrected (4Å)
Elevation
Azimuth
# of images
FSC (Model-map)
FSC = 0.5
3.43 Å
FSC = 0.143
2.25 Å
Resolution (Å)

b

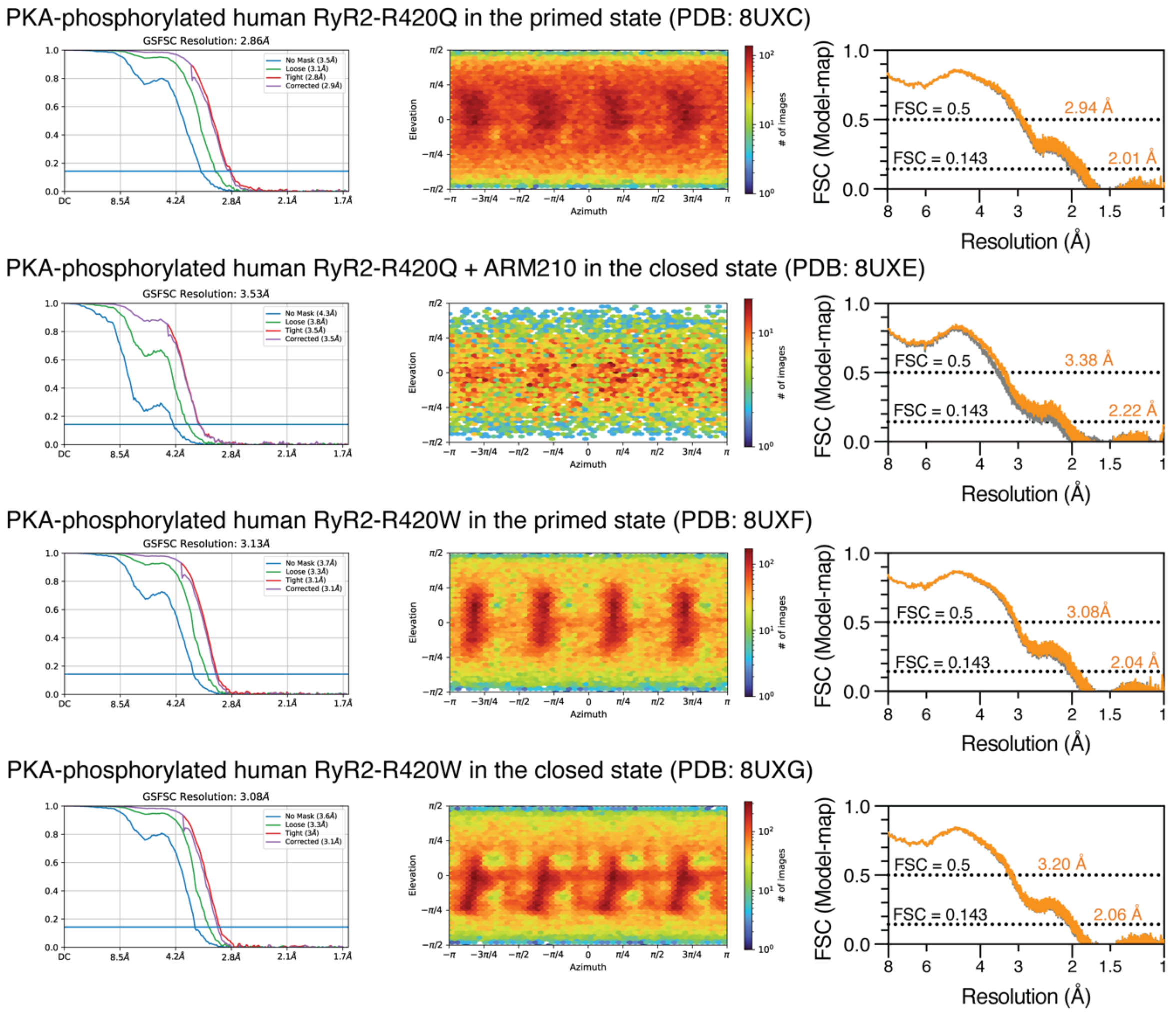
PKA-phosphorylated human RyR2-R420Q in the primed state (PDB: 8UXC)
GSFSC Resolution: 2.86Å
No Mask (3.5Å)
Loose (3.1Å)
Tight (2.8Å)
Corrected (2.9Å)
FSC = 0.5
2.94 Å
FSC = 0.143
2.01 Å
PKA-phosphorylated human RyR2-R420Q + ARM210 in the closed state (PDB: 8UXE)
GSFSC Resolution: 3.53Å
No Mask (4.3Å)
Loose (3.8Å)
Tight (3.5Å)
Corrected (3.5Å)
FSC = 0.5
3.38 Å
FSC = 0.143
2.22 Å
PKA-phosphorylated human RyR2-R420W in the primed state (PDB: 8UXF)
GSFSC Resolution: 3.13Å
No Mask (3.7Å)
Loose (3.3Å)
Tight (3.1Å)
Corrected (3.1Å)
FSC = 0.5
3.08Å
FSC = 0.143
2.04 Å
PKA-phosphorylated human RyR2-R420W in the closed state (PDB: 8UXG)
GSFSC Resolution: 3.08Å
No Mask (3.6Å)
Loose (3.3Å)
Tight (3Å)
Corrected (3.1Å)
FSC = 0.5
3.20 Å
FSC = 0.143
2.06 Å
Elevation
Azimuth
# of images
FSC (Model-map)
Resolution (Å)

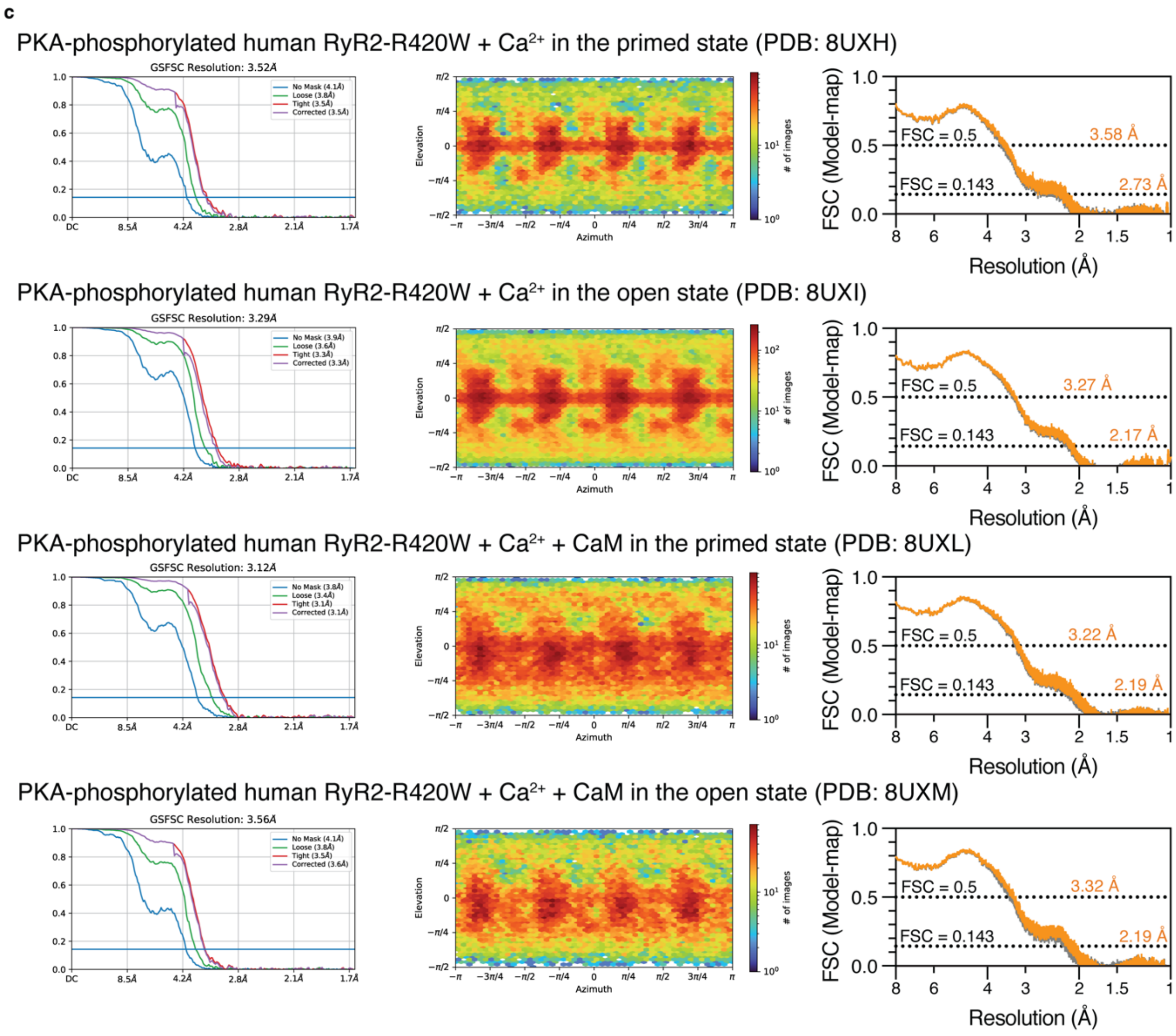

Supplementary Fig. 3. FSC curves. a-c The GSFSCs (left) and the viewing angle distributions (middle) of the global non-uniform refinement performed in cryoSPARC before any local refinement, and the FSC model-map performed in PHENIX (right) for each structure are shown.

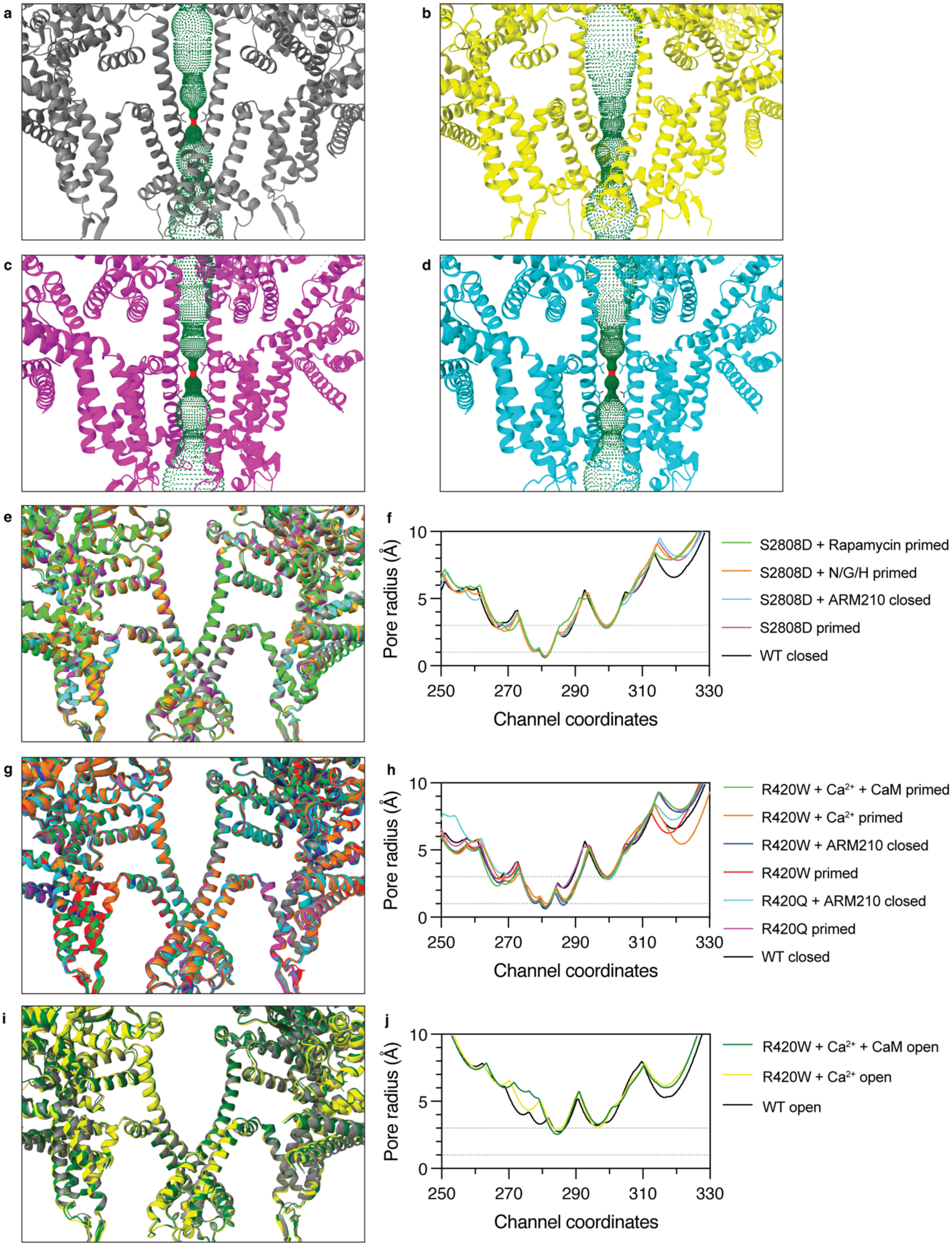

a
b
c
d
e
f
Pore radius (Å)
Channel coordinates
10
5
0
250
270
290
310
330
S2808D + Rapamycin primed
S2808D + N/G/H primed
S2808D + ARM210 closed
S2808D primed
WT closed
g
h
Pore radius (Å)
Channel coordinates
R420W + Ca2+ + CaM primed
R420W + Ca2+ primed
R420W + ARM210 closed
R420W primed
R420Q + ARM210 closed
R420Q primed
WT closed
i
j
Pore radius (Å)
Channel coordinates
R420W + Ca2+ + CaM open
R420W + Ca2+ open
WT open

Supplementary Fig. 4. Pore analyses of all structures. a-d Atomic models overlapped with the permeation path calculated using HOLE (illustrated as green dots for diameter greater than 2 Å and red dots for diameter less than 2 Å) for closed PKA-phosphorylated WT RyR2 (**a**), open PKA-phosphorylated WT RyR2 (**b**), primed PKA-phosphorylated RyR2-R420Q (**c**), and closed PKA-phosphorylated RyR2-R420Q + ARM210 (D). **e** Aligned atomic models of all closed/primed structures focusing on the pore and TM domain. **f** Pore radii estimation for each structure calculated with HOLE. Channel coordinates are arbitrary. Atomic model alignment and HOLE analyses suggest that no significant differences are found in the pore of the closed states. Only the sidechain of residue Q4863 seems to fluctuate, but probably because of the weak cryo-EM density resulting from the expected dynamic behavior of the solvent-exposed polar sidechain. **g-j** same as **e,f** but with the RyR2-S2808D **(g,h)** and the RyR2-R420W open states **(i,j)**.

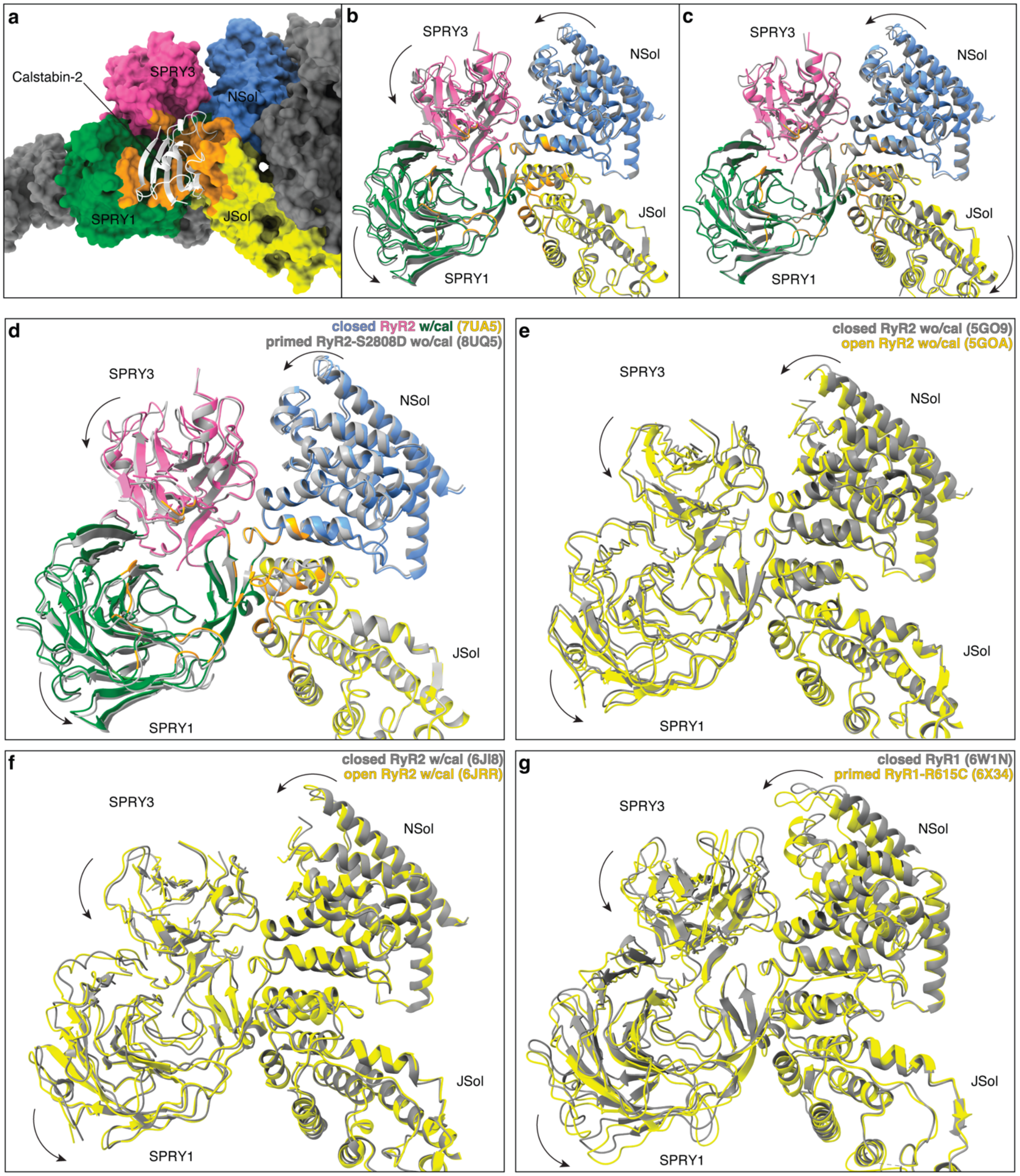

Supplementary Fig. 5. Calstabin binding site in different models. a Atomic model of RyR2 showing calstabin-2 (white ribbons), calstabin-2 interacting RyR2 domains (NSol in light blue, SPRY1 in green, SPRY3 in pink, and JSol in yellow), and RyR2-calstabin-2 interacting surface (orange). **b,c** Atomic models of the calstabin-2 interacting RyR2 domains in the closed state

(PDB:7UA5, colors) and in the open state (PDB: 7UA9, grey). Models are aligned on the JSol (**b**) and SPRY1-3 (**c**) domains. Relative inter-domain motions are shown with arrows. **d** Atomic models of the calstabin-2 interacting RyR2 domains of human WT RyR2 in the closed state (PDB:7UA5, colors) and RyR2-S2808D in the primed state without calstabin-2 (PDB:8UQ5, grey). Models are aligned in the JSol. Relative inter-domain motions are shown with arrows. **e** Same as **d** but of pig RyR2 in the closed state without calstabin-2 (PDB:5GO9, grey) and pig RyR2 in the open state without calstabin-2 (PDB:5GOA, yellow). **f** Same as **d** but of pig RyR2 in the closed state with calstabin-2 (PDB:6JI8, grey) and pig RyR2 in the open state with calstabin-2 (PDB:6JRR, yellow). **g** Same as **d** but of pig RyR1 in the closed state (PDB:6W1N, grey) and pig RyR1-R615C in the primed state (PDB:6X34, yellow).

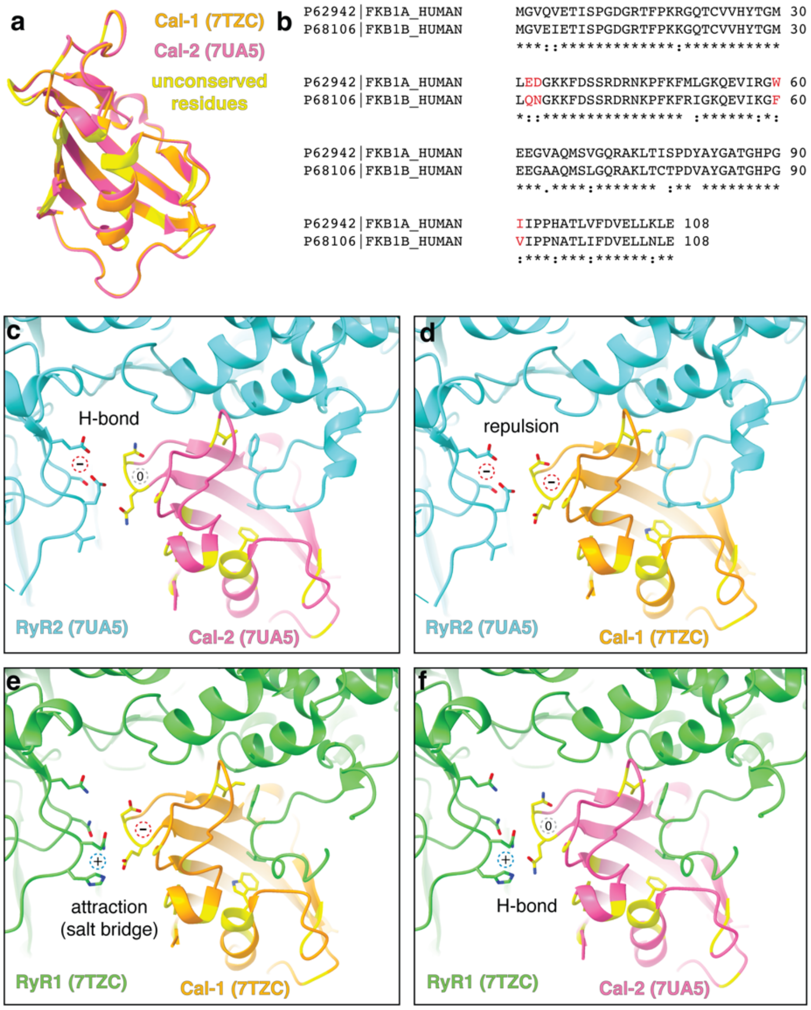

Supplementary Fig. 6. Structures of the complexes between RyRs and calstabins. a Overlapped atomic models of calstabin-1 (PDB: 7TZC, orange) and calstabin-2 (PDB: 7UA5, magenta) showing their almost identical backbone structure. Unconserved residues are colored (yellow). **b** Sequence alignment of human calstabin-1 (FKBP1A) and calstabin-2 (FKBP1B). Only the four uncoserved residues that interact with RyRs are colored (red). **c-f** Atomic models of RyR2 and calstabin-2 **(c)**, RyR2 and calstabin-1 **(d)**, RyR1 and calstabin-1 **(e)**, and RyR1 and calstabin-2 **(f)**. Models are aligned on the calstabins models. Potential interaction (H-bond, repulsion, and attraction) and potential interacting residues between RyRs and casltabins are shown for RyR1 (N1296, Q1299, H1300, and N1545), RyR2 (E1309 and E1312), calstabin-1 (E32 and D33), and calstabin-2 (Q32 and N33).

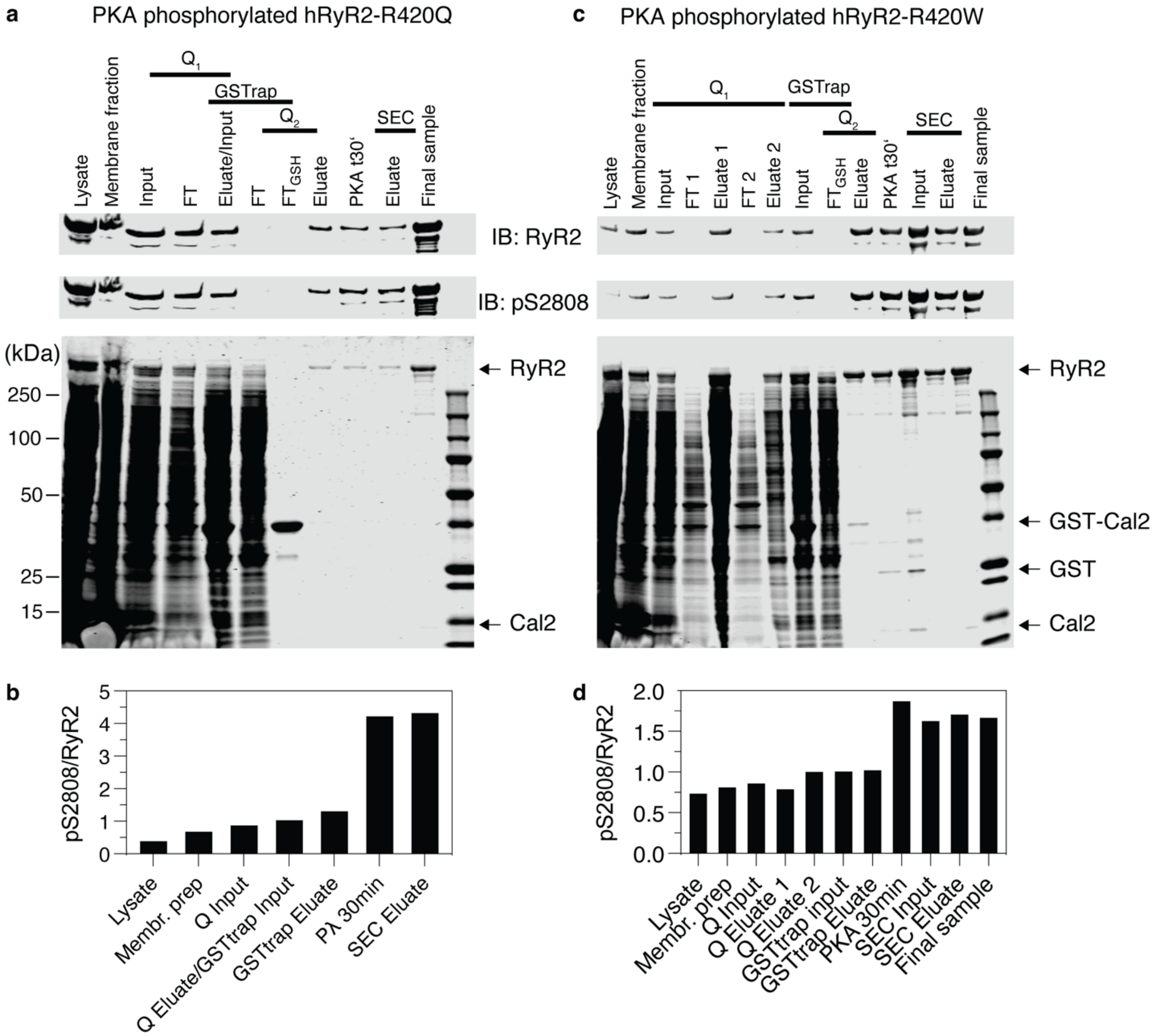

Supplementary Fig. 7. Purification of recombinant human RyR2. a,c Immunoblot (top) and SDS-PAGE (bottom) of purification of human recombinant RyR2-R420Q (**a**) and RyR2-R420W (**c**) expressed in HEK293 cells. **b,d** Ratio of normalized intensities of the pS2808 and total RyR2 bands. After PKA phosphorylation, the intensity increases ~3-fold (**b**) and ~2-fold (**d**), reaching a saturation point.

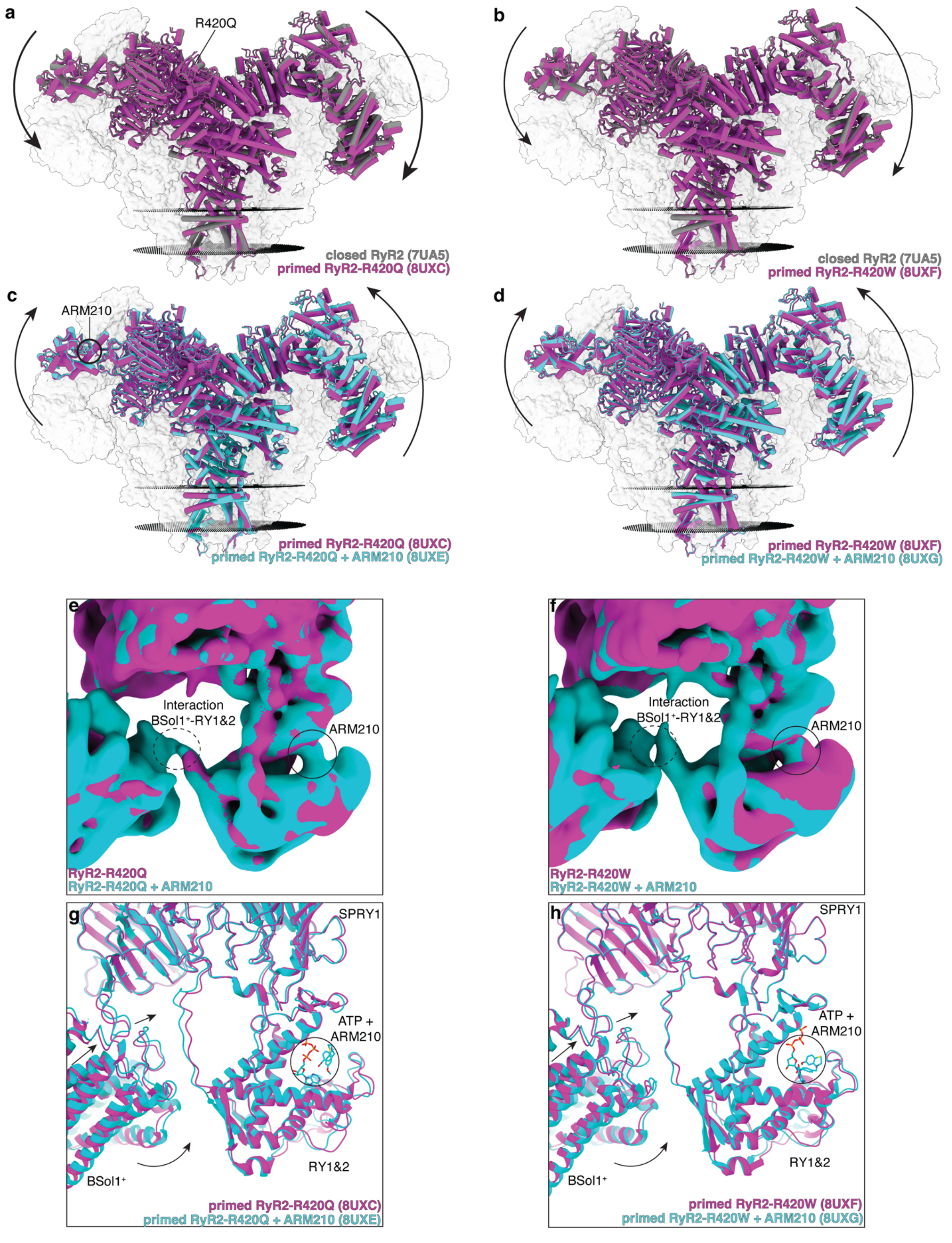

a
R420Q
closed RyR2 (7UA5)
primed RyR2-R420Q (8UXC)
b
closed RyR2 (7UA5)
primed RyR2-R420W (8UXF)
c
ARM210
primed RyR2-R420Q (8UXC)
primed RyR2-R420Q + ARM210 (8UXE)
d
primed RyR2-R420W (8UXF)
primed RyR2-R420W + ARM210 (8UXG)
e
Interaction BSol1⁺-RY1&2
ARM210
RyR2-R420Q
RyR2-R420Q + ARM210
f
Interaction BSol1⁺-RY1&2
ARM210
RyR2-R420W
RyR2-R420W + ARM210
g
SPRY1
ATP + ARM210
BSol1⁺
RY1&2
primed RyR2-R420Q (8UXC)
primed RyR2-R420Q + ARM210 (8UXE)
h
SPRY1
ATP + ARM210
BSol1⁺
RY1&2
primed RyR2-R420W (8UXF)
primed RyR2-R420W + ARM210 (8UXG)

Supplementary Fig. 8. Cryo-EM reconstructions of human RyR2 shows that the mutation-induced "primed" state is reversed by treatment with the Rycal ARM210. a Overlapped models of closed PKA-phosphorylated RyR2 (grey) and primed PKA-phosphorylated RyR2-R420Q (magenta). The arrows show that the cytoplasmic shell of the channel shifts downward-outward when going from the closed to the primed state. **b** Same as **a** but with primed PKA-phosphorylated RyR2-R420W (magenta). **c** Overlapped models of primed PKA-phosphorylated RyR2-R420Q (magenta) and closed PKA-phosphorylated RyR2-R420Q in the presence of ARM210 (cyan). The arrows show that the cytoplasmic shell of the channel shifts upward-inward when going from the primed to the closed state. **d** Same as **c** but with primed PKA-phosphorylated RyR2-R420W (magenta) and closed PKA-phosphorylated RyR2-R420W + ARM210 (cyan). **e** Cryo-EM maps of local refinement cryoSPRAC jobs of primed PKA-phosphorylated RyR2-R420Q (magenta), and closed PKA-phosphorylated RyR2-R420Q + ARM210 (cyan). For better interpretation, a softening gaussian was applied to the cryo-EM maps. Interaction between BSol1 and RY1&2, and ARM210 densities are labelled. **f** Same as **e** but with primed PKA-phosphorylated RyR2-R420W (magenta), and closed PKA-phosphorylated RyR2-R420W + ARM210 (cyan). **g** Aligned models of primed PKA-phosphorylated RyR2-R420Q (magenta), and closed PKA-phosphorylated RyR2-R420Q + ARM210 (cyan). Conformational changes of the RY1&2 and BSol domains are shown with arrows. ATP + ARM210 are labelled. **h** Same as **g** but with primed PKA-phosphorylated RyR2-R420W (magenta), and closed PKA-phosphorylated RyR2-R420W + ARM210 (cyan).

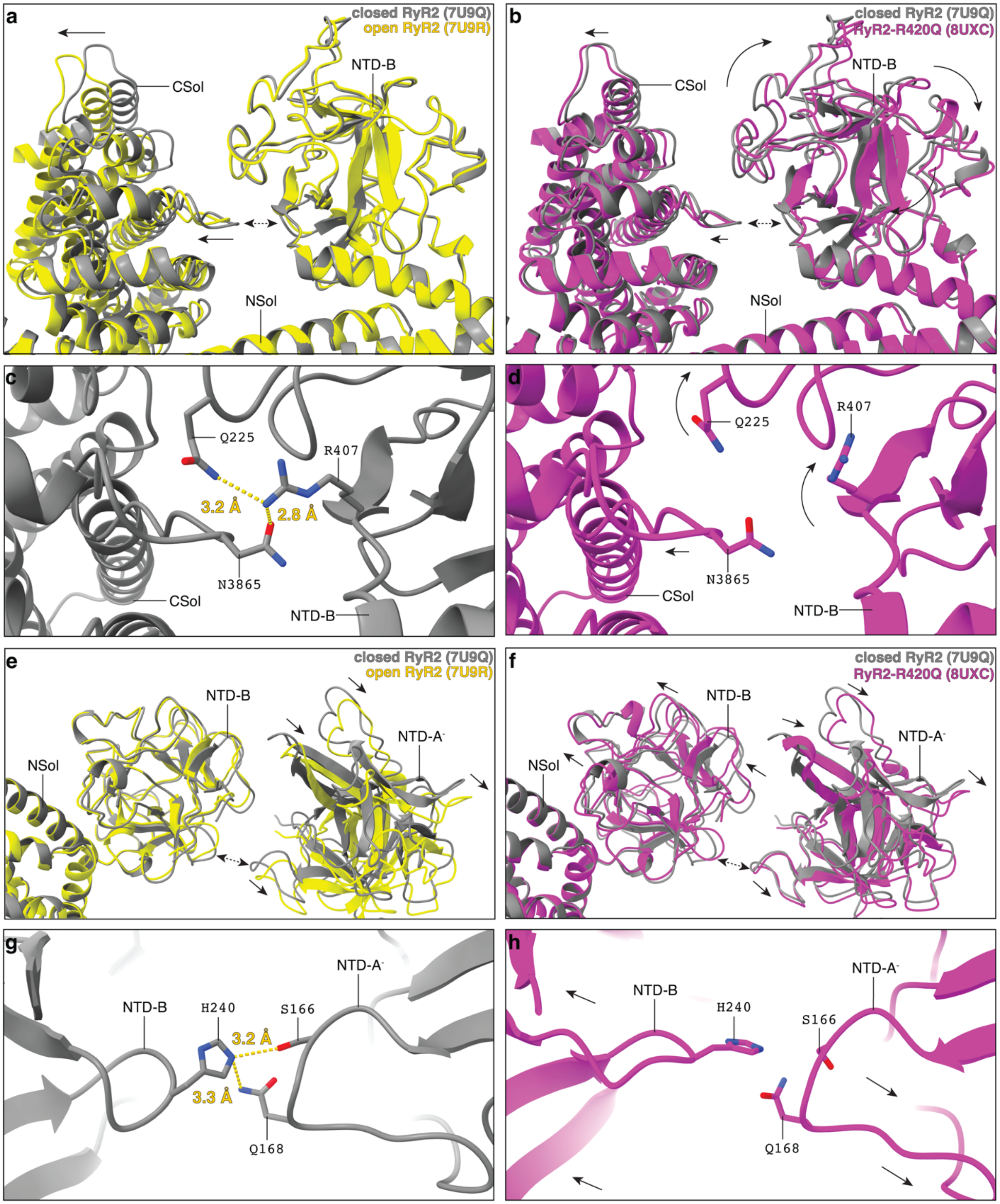

Supplementary Fig. 9. R420Q-induced rotation of the NTD-B domain disrupts interaction with the CSol and NTD-A* domains. a-d Aligned models focused on the NTD-B and CSol domains of closed PKA-phosphorylated WT RyR2 (PDB:7U9Q, grey), open PKA-phosphorylated WT RyR2 (PDB:7U9R, yellow), and primed PKA-phosphorylated RyR2-R420Q (PDB:8UXC,

magenta). Models were aligned at the NSol domain. Interdomain interactions are shown (dashed double arrow). Conformational changes are shown with arrows. Residues and distances stabilizing interdomain interactions are labelled. **e-h** Same as **a-d** but focusing on the interaction between the NTD-B and NTD-A$^-$ domains. NTD-A$^-$ stands for the NTD-A domain of the neighbor protomer.

Variant	Onset age			koff	Kd	kon
	Average	SD	Count			
Arg2474Ser/Gly	7.4	4.5	8	0.095	232	407370
Ser2246Leu	8.7	4.8	18	0.076	185	410636
Arg420Gln	14.1	11.1	27	0.075	160	469200
Arg4959Gln	14.9	10.0	12	0.079	183	430215
Arg420Trp	20.1	7.3	12	0.071	168	407370
Glu4076Lys	29.7	20.6	3	0.061	151	400362
Arg3570Trp	36.0	26.9	2	0.064	155	415632
WT	60.0	*	*	0.043	105	406991

Supplementary Fig. 10. Calstabin binding site in different models. Table of the analyzed variants from the database,[53] showing average, standard deviation (SD), and number of patients (count) of onset age and diagnosis age. Also, included the k_{on} ($M^{-1}.min^{-1}$), k_{off} (min^{-1}), and K_d (nM) of the recombinant RyR2 variants expressed in HEK293 cells.

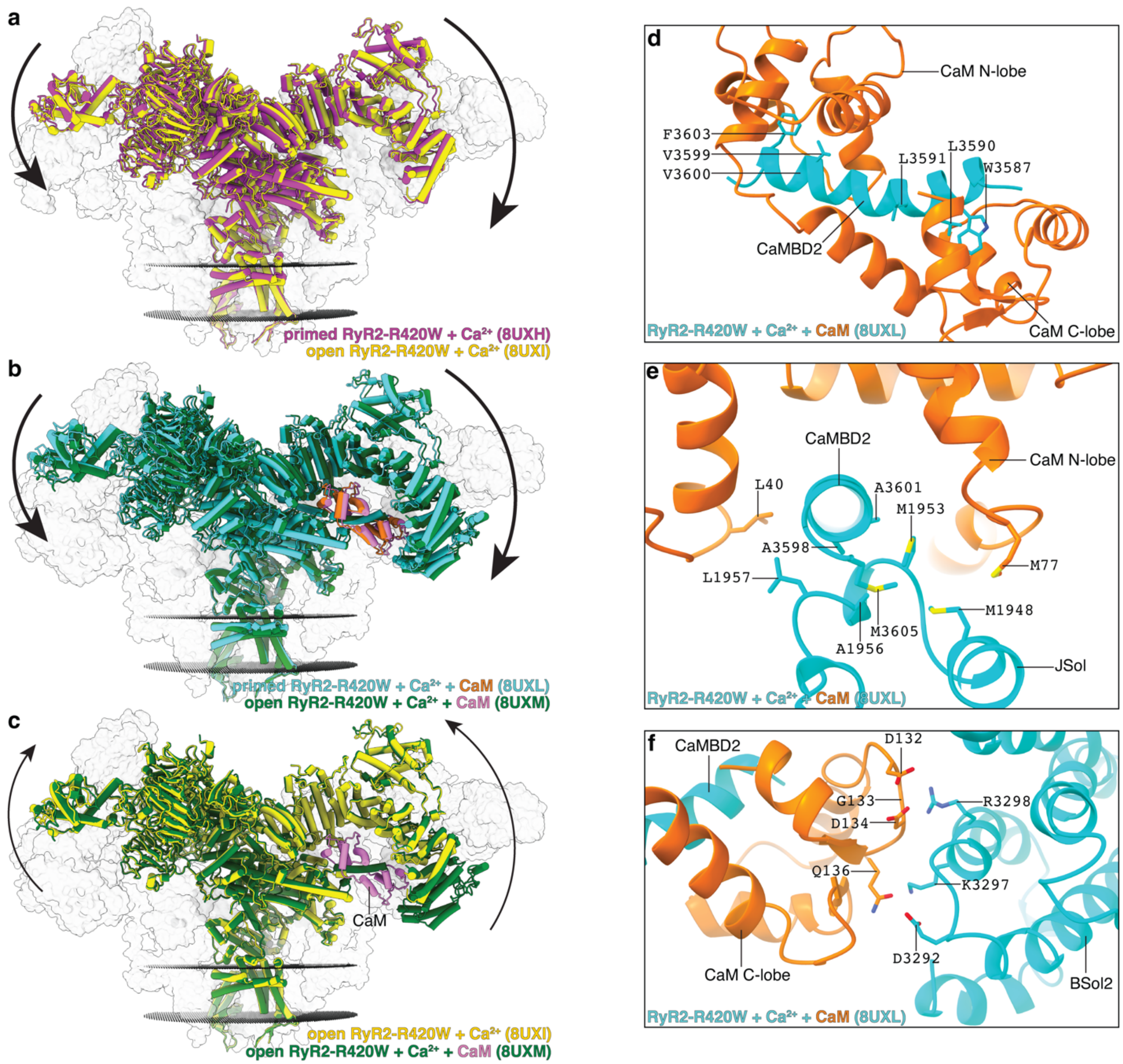

Supplementary Fig. 11. RyR2-R420W increased sensitivity to Ca^{2+} is partially reversed by accesory protein CaM. a Overlapped models of primed PKA-phosphorylated RyR2-R420W + Ca^{2+} (PDB:8UXH, magenta) and open PKA-phosphorylated RyR2-R420W + Ca^{2+} (PDB:8UXI, yellow). The arrows show that the cytoplasmic shell of the channel shifts downward-outward when going from the primed to the open state. To facilitate visualization only the front protomer is shown in colors, while the other three protomers are shown as grey transparent volumes. The positions of the sarcoplasmic reticulum membranes are shown as black discs. **b** Overlapped models of primed PKA-phosphorylated RyR2-R420W + Ca^{2+} +CaM (PDB:8UXL, cyan) and open PKA-phosphorylated RyR2-R420W + Ca^{2+} + CaM (PDB:8UXM, green). The arrows show that the

cytoplasmic shell of the channel shifts downward-outward when going from the primed to the open state, even in the presence of CaM. **c** Overlapped models of open PKA-phosphorylated RyR2-R420W + Ca^{2+} (PDB:8UXI, yellow) and open PKA-phosphorylated RyR2-R420W + Ca^{2+} + CaM (PDB:8UXM, green). The arrows show that the cytoplasmic shell of the channel shifts upward-inward in the presence of CaM, suggesting CaM has a stabilizing effect even in the open state. **d-f** Different views of the model of primed PKA-phosphorylated RyR2-R420W + Ca^{2+} + CaM (PDB:8UXL) focused on the CaM binding site. Residues involved in protein-protein interactions and interdomain interactions are labelled.

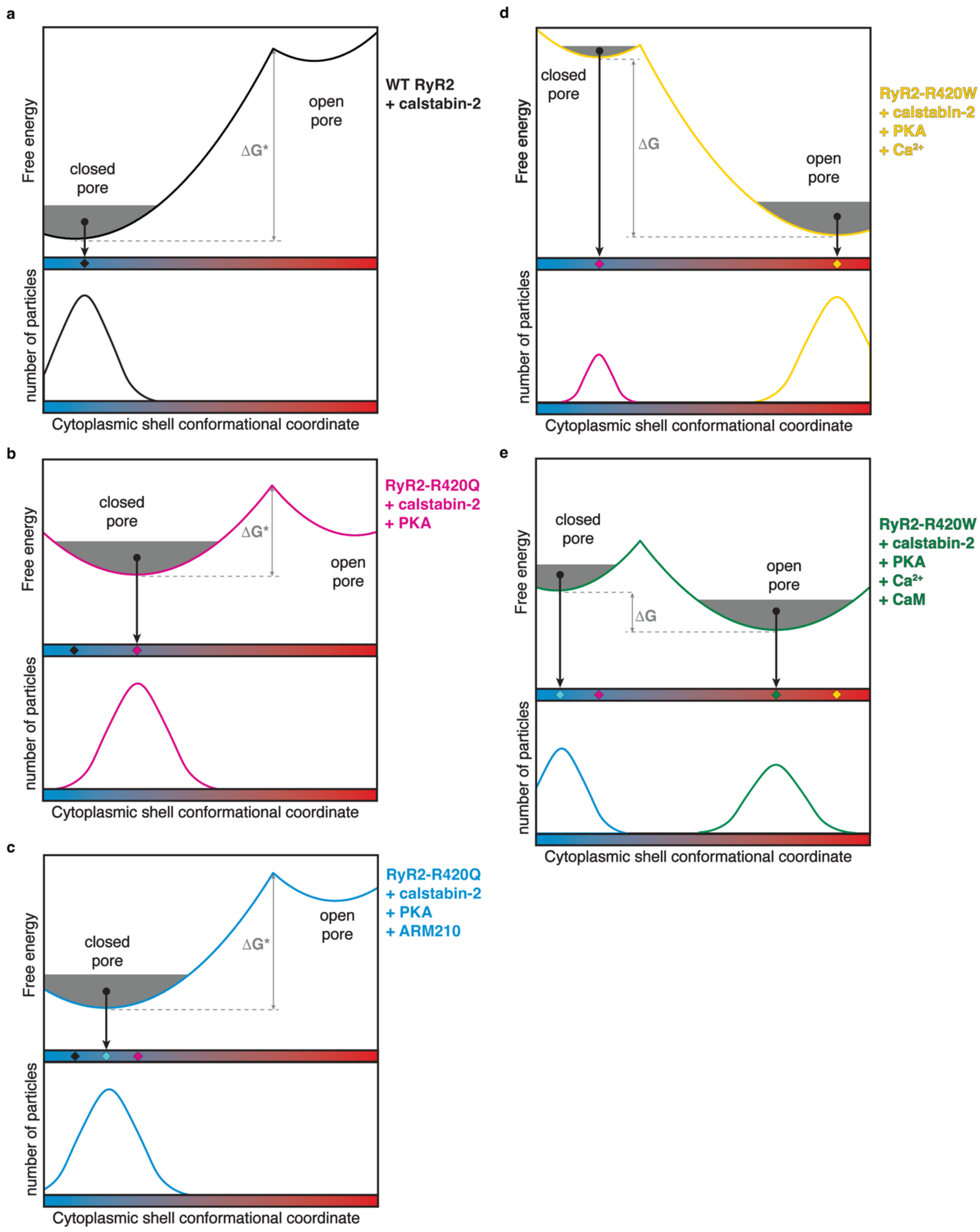

Supplementary Fig. 12. The proposed energy landscape explains the conformations and populations observed by cryo-EM. a-c Schematic view of the dynamic energy landscape of conformational space of the cytoplasmic shell for WT RyR2 + calstabin-2 (black, **a**), PKA

phosphorylated RyR2-R420Q + calstabin-2 (magenta, **b**), and PKA phosphorylated RyR2-R420Q + calstabin-2 + ARM210 (cyan, **c**). Energy landscapes were estimated using the approach from Figure 5. The grey area represents the conformations populated at a level of energy set by conditions and temperature. The average conformation is showed as a dot and projected to the conformational axis, which is observed in cryo-EM. The relative position of the average conformations corelates to the average conformations observed in cryo-EM (black > cyan > magenta). The population in the open state is very low and undetectable by cryo-EM. However, bilayer experiments suggest the channels visit the open state, with an increased open probability in the CPVT channels. The increased open probability is explained here by a decreased of the activation energy (ΔG^*) compared to the WT and the ARM210 treated channels. **d,e** Same as **a** but with PKA phosphorylated RyR2-R420W + calstabin-2 + Ca^{2+} (yellow, **d**) and PKA phosphorylated RyR2-R420W + calstabin-2 + Ca^{2+} + CaM (green, **e**). In this case both the open and closed states are present in cryo-EM. The change in the energy landscape explains the upward-inward movements observed for the cryo-EM structures. In addition, the lower net energy difference (ΔG) explains the shift in population observed in the cryo-EM particles.

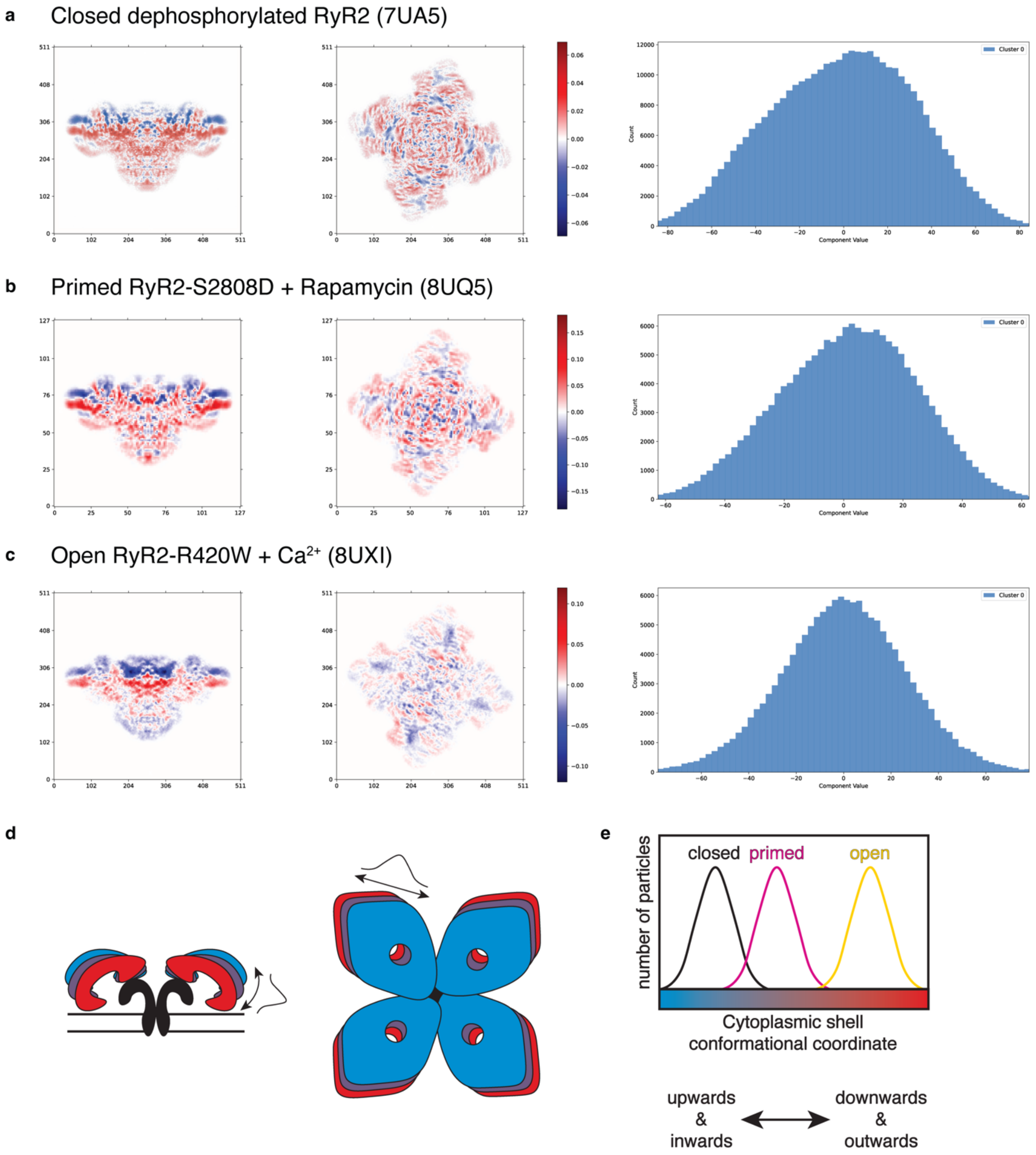

Supplementary Fig. 13. 3D Variability Analysis of RyR2 variants. a-c Principal mode of variability and particle count showing normal distribution for closed dephosphorylated RyR2 **(a)**, primed RyR2-S2808D + Rapamycin **(b)**, and open RyR2-R420W + Ca^{2+} **(c)**. **d** Schematic model of normal dynamics of RyR2 from the side and top views. **e** Representation of the cryo-EM particle count and the conformation of the cytoplasmic shell. Supplementary Figure 13d adapted from Marco C. Miotto et al., Structural analyses of human ryanodine receptor type 2 channels reveal the mechanisms for sudden cardiac death and treatment. Sci. Adv. 8,eabo1272(2022).DOI:10.1126/sciadv.abo1272 under a CC BY license: https://creativecommons.org/licenses/by/4.0/.[10]

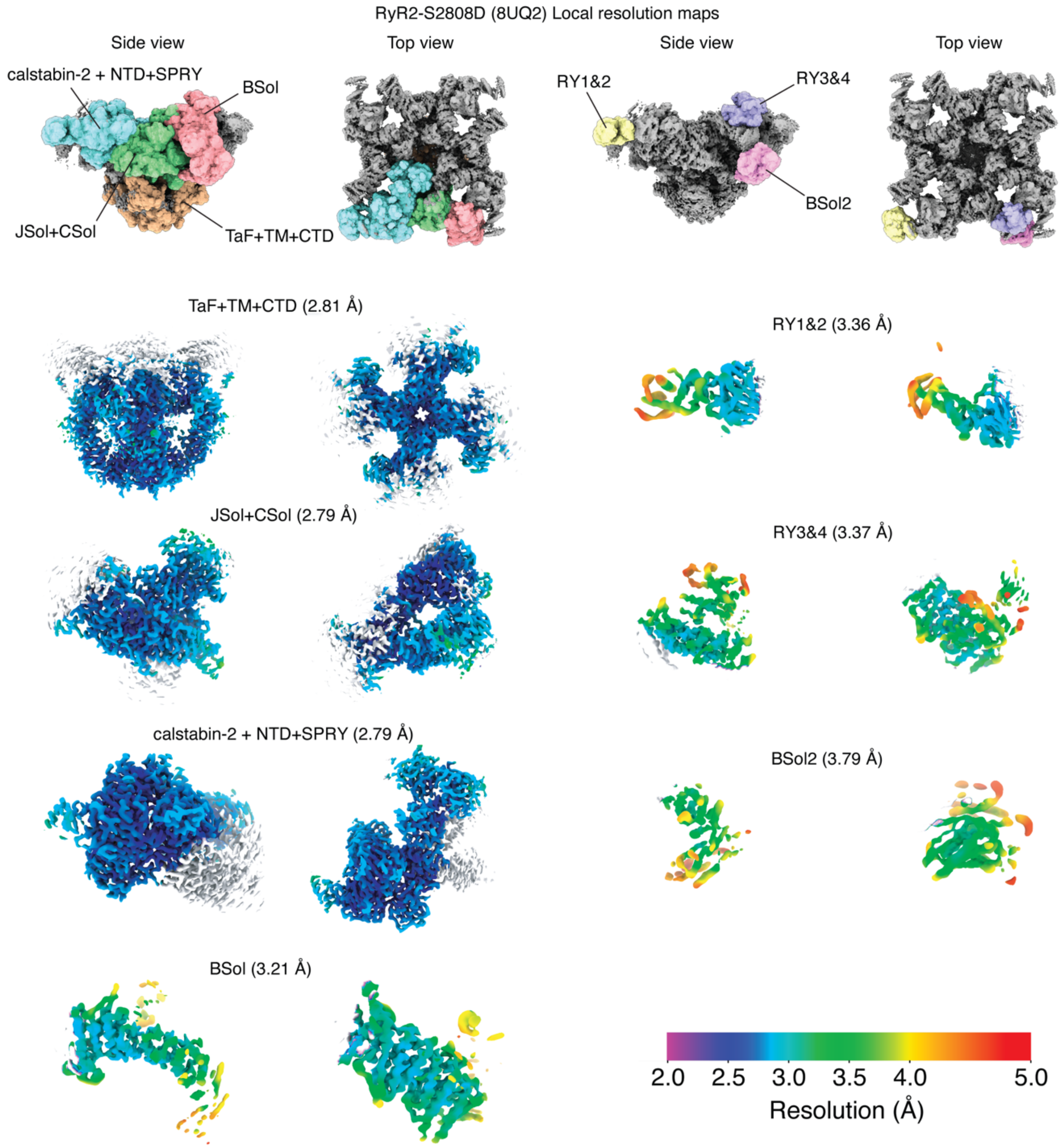

Supplementary Fig. 14. Representative local resolution maps. Local refinement cryo-EM maps colored by local resolution shown for the RyR2-R2808D (8UQ2).

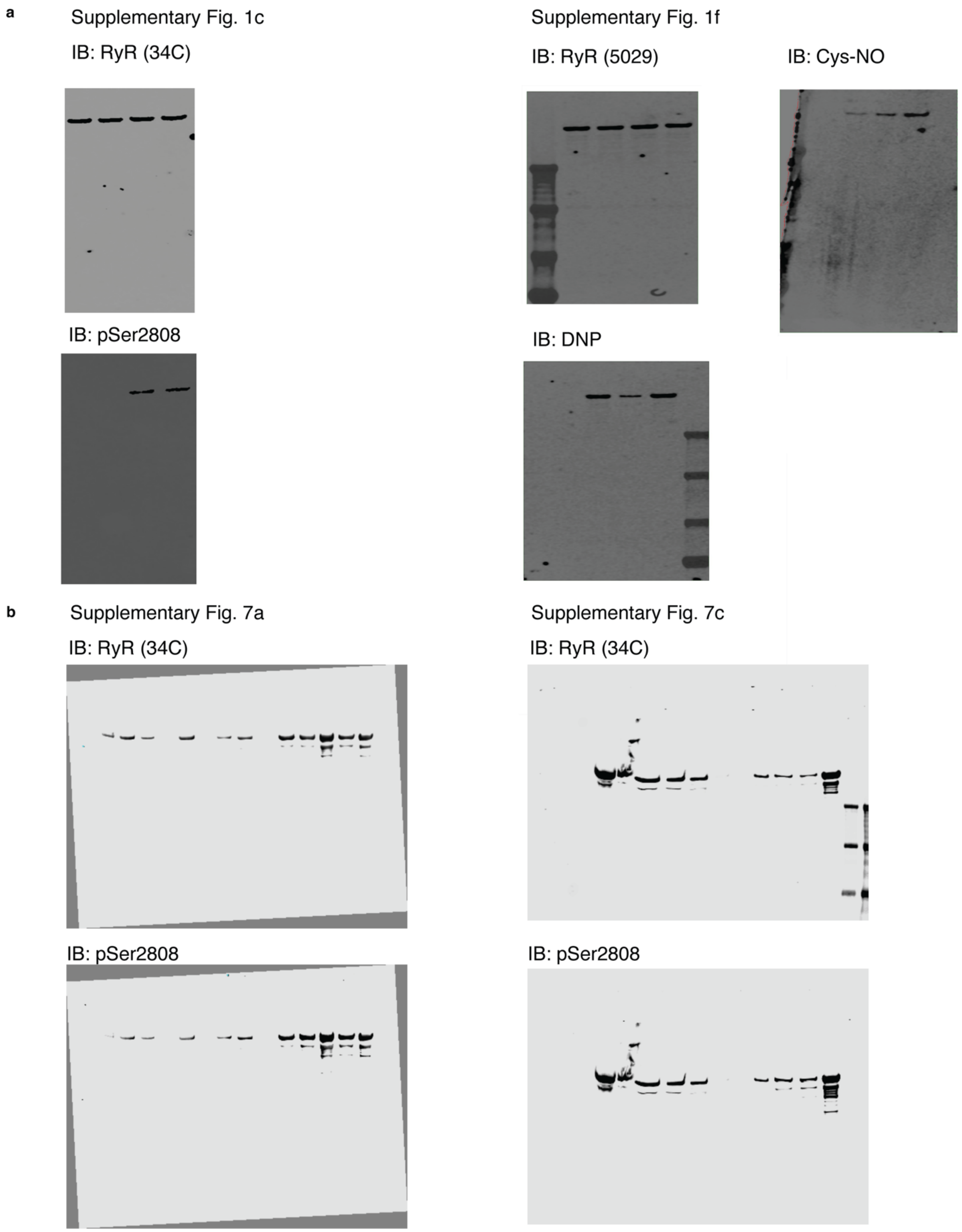

Supplementary Fig. 15. Uncropped western blots. a Uncropped western blots for supplementary figure 1. **b** Uncropped western blots for supplementary figure 7.

Supplementary Table 1. Domain span for human RyR2.

Identification	Symbol	Residue Span
Cytoplasmic shell		(1-3633)
N-terminal domain	NTD	(1-639)
N-terminal domain A	NTD-A	(1-219)
N-terminal domain B	NTD-B	(220-408)
N-terminal solenoid	NSol	(409-639)
SPRY domain	SPRY	(640-1646)
SP1a/ryanodine receptor domain 1	SPRY1	(640-861,1463-1483,1595-1646)
SP1a/ryanodine receptor domain 2	SPRY2	(1076-1255)
SP1a/ryanodine receptor domain 3	SPRY3	(1256-1462,1484-1594)
RYR repeats 1 & 2	RY1&2	(862-1076)
Junctional solenoid	JSol	(1647-2108)
Bridging solenoid	BSol	(2109-3564)
Bridging solenoid 1	BSol1	(2109-2681,2916-3042)
Bridging solenoid 2	BSol2	(3042-3344)
Bridging solenoid 3	BSol3	(3345-3564)
RYR repeats 3 & 4	RY3&4	(2682-2915)
Shell-core linker peptide	SCLP	(3565-3633)
Activation core and pore		(3634-4967)
Core solenoid	CSol	(3634-4130)
EF-hand pair	EF1&2	(4016-4090)
Thumb and forefingers domain	TaF	(4131-4209)
Transmembrane domain	TM	(4237-4886)
Auxiliary intramembrane helices	Sx	(4237-4310)
Pseudo voltage sensor domain	pVSD	(4480-4750)
Channel pore domain	Pore	(4751-4886)
C-terminal domain	CTD	(4887-4967)
Zn-finger domain	ZnF	(4887-4914)

Supplementary Table 2. Cryo-EM Statistics.

Sample	RyR2-S2808D	RyR2- S2808D + ARM210	RyR2- S2808D + NOC-12/GSH	RyR2- S2808D + Rapamycin
State	primed	closed	primed	primed
PDB ID	8UQ2	8UQ3	8UQ4	8UQ5
EMDB ID	EMD-42458	EMD-42459	EMD-42460	EMD-42461
**Data collection**				
Microscope	FEI Titan Krios	FEI Titan Krios	FEI Titan Krios	FEI Titan Krios
Detector	Gatan K3	Gatan K3	Gatan K3	Gatan K3
Voltage (kV)	300	300	300	300
Magnification	105,000	105,000	105,000	105,000
Exposure ($e^-/Å^2$)	58	58	58	58
Defocus range (μm)	-0.4 to -1.2	-0.4 to -1.2	-0.4 to -1.2	-0.4 to -1.2
Pixel size (Å)	0.83	0.83	0.83	0.83
**Processing**				
Software	cryoSPARC	cryoSPARC	cryoSPARC	cryoSPARC
Symmetry	C4	C4	C4	C4
Initial particles (N)	267,550	137,303	83,074	320,951
Final particles (N)	191,898	83,460	19,753	18,232
Global map resolution (Å)*	2.98	3.18	3.64	3.96
Local maps resolution range (Å)[‡]	2.79-3.79	2.92-4.16	3.22-5.63	3.11-5.62
**Model Composition**				
Peptide chains	8	8	8	4
Nonhydrogen	138,608	138,700	138,608	135,336
Protein residues	17,324	17,324	17,324	16,896
Ligands	12	16	12	12
**Mean *B* factors ($Å^2$)**				
Protein	112.09	120.82	151.90	141.97
Ligands	161.25	179.93	208.42	192.77
**RMSD**				
Bond length (Å)	0.003	0.003	0.003	0.003
Bond angles (°)	0.746	0.732	0.752	0.720
**Ramachandran**				
Favored (%)	97.16	97.04	96.58	97.31
Allowed (%)	2.77	2.92	3.33	2.60
Disallowed (%)	0.07	0.05	0.09	0.10
**Validation**				
MolProbity score	1.72	1.77	1.90	1.72
Clashscore	11.30	12.18	15.03	11.81
Rotamer outliers (%)	0.50	0.18	0.24	0.30
FSC model 0.5 (Å)[#]	2.99	3.13	3.53	3.43

Supplementary Table 2 continuation.

Sample	RyR2-R420Q	RyR2-R420Q +ARM210	RyR2-R420W	RyR2-R420W + ARM210
State	primed	closed	primed	closed
PDB ID	8UXC	8UXE	8UXF	8UXG
EMDB ID	EMD-42759	EMD-42761	EMD-42762	EMD-42763
**Data collection**				
Microscope	FEI Titan Krios	FEI Titan Krios	FEI Titan Krios	FEI Titan Krios
Detector	Gatan K3	Gatan K3	Gatan K3	Gatan K3
Voltage (kV)	300	300	300	300
Magnification	105,000	105,000	105,000	105,000
Exposure (e^-/Å^2)	58	58	58	58
Defocus range (μm)	-0.4 to -1.2	-0.4 to -1.2	-0.4 to -1.2	-0.4 to -1.2
Pixel size (Å)	0.83	0.83	0.83	0.83
**Processing**				
Software	cryoSPARC	cryoSPARC	cryoSPARC	cryoSPARC
Symmetry	C4	C4	C4	C4
Initial particles (N)	187,859	20,900	115,505	206,663
Final particles (N)	143,933	14,924	102,478	181,724
Global map resolution (Å)*	2.86	3.53	3.13	3.08
Local maps resolution range (Å)[‡]	2.64-3.35	3.10-5.02	2.90-3.74	2.91-3.38
**Model Composition**				
Peptide chains	8	8	8	8
Nonhydrogen	138,600	138,692	138,620	138,712
Protein residues	17,324	17,324	17,324	17,324
Ligands	12	16	12	16
**Mean *B* factors (Å^2)**				
Protein	110.19	129.90	105.51	110.57
Ligands	147.54	258.08	171.76	165.53
**RMSD**				
Bond length (Å)	0.003	0.004	0.004	0.003
Bond angles (°)	0.672	0.713	0.697	0.720
**Ramachandran**				
Favored (%)	97.38	96.83	97.62	97.67
Allowed (%)	2.55	3.05	2.34	2.28
Disallowed (%)	0.07	0.12	0.05	0.05
**Validation**				
MolProbity score	1.61	1.81	1.62	1.58
Clashscore	9.29	12.83	10.46	9.75
Rotamer outliers (%)	0.03	0.37	0.58	0.74
FSC model 0.5 (Å)[#]	2.94	3.38	3.08	3.20

Supplementary Table 2 continuation.

Sample	RyR2-R420W + Ca^{2+}		RyR2-R420W + Ca^{2+} + CaM	
State	primed	open	primed	open
PDB ID	8UXH	8UXI	8UXL	8UXM
EMDB ID	EMD-42764	EMD-42765	EMD-42768	EMD-42769
**Data collection**				
Microscope	FEI Titan Krios		FEI Titan Krios	
Detector	Gatan K3		Gatan K3	
Voltage (kV)	300		300	
Magnification	105,000		105,000	
Exposure (e^-/Å^2)	58		58	
Defocus range (μm)	-0.4 to -1.2		-0.4 to -1.2	
Pixel size (Å)	0.83		0.83	
**Processing**				
Software	cryoSPARC		cryoSPARC	
Symmetry	C4		C4	
Initial particles (N)	254,828		134,776	
Final particles (N)	55,886	149,448	77,625	49,606
Global map resolution (Å)*	3.52	3.29	3.12	3.56
Local maps resolution range (Å)‡	3.15-4.11	3.01-3.82	2.88-5.03	3.02-6.01
**Model Composition**				
Peptide chains	8	8	12	12
Nonhydrogen	131,656	131,656	143,500	143,500
Protein residues	16,444	16,444	17,928	17,928
Ligands	16	16	32	32
**Mean *B* factors (Å^2)**				
Protein	158.66	130.95	129.59	134.13
Ligands	338.76	234.58	197.73	205.78
**R.m.s. deviations**				
Bond length (Å)	0.004	0.003	0.004	0.011
Bond angles (°)	0.854	0.825	0.881	0.751
**Ramachandran**				
Favored (%)	97.05	96.77	96.82	97.09
Allowed (%)	2.85	3.15	3.09	2.82
Disallowed (%)	0.10	0.07	0.09	0.09
**Validation**				
MolProbity score	1.94	1.90	1.92	1.75
Clashscore	13.01	12.67	13.79	11.89
Rotamer outliers (%)	1.58	1.28	1.25	0.74
FSC model 0.5 (Å)#	3.58	3.27	3.22	3.32

* Map resolution of the non-uniform refinement determined by CryoSPARC before local masks.

‡ Map resolution range represents the range determined by local refinements in cryoSPARC using the masks described in Methods.

# Value obtained from Phenix validation tool.
