# Supplementary material for: Structural basis for ryanodine receptor type 2 leak in heart failure and arrhythmogenic disorders
Source: Nat Commun. 2024 Sep 15;15:8080. doi: 10.1038/s41467-024-51791-y (PMC11402997; doi:10.1038/s41467-024-51791-y)
Supplement: Supplementary file 3 — Description of Additional Supplementary Files [file 41467_2024_51791_MOESM3_ESM.pdf]

**Description of Additional Supplementary Files**

**File name: Supplementary Movie 1**

**Description:** Atomic model of the N-terminal domain of RyR2 (PDB 7UA5), showing the residues R417 (yellow) and R420 (red) surrounded by the NTD-A (blue), NTD-B (light blue), and NSol (grey) domains

**File name: Supplementary Movie 2**

**Description:** Atomic model of primed RyR2-R420W + Ca2+ + CaM (PBD 8UXL) overlapped with its respective cryo-EM map, showing the residues in the interface between RyR2-R420W (cyan) and CaM (orange).

**File name: Supplementary Movie 3**

**Description:** Cryo-EM volumes of the 3D Variability Analysis RyR2-S2808D showing the movement of the cytoplasmic shell from the most upward-inward conformation (cyan) to the most downward-outward conformation (red) from the side view.

**File name: Supplementary Movie 4**

**Description:** Cryo-EM volumes of the 3D Variability Analysis RyR2-S2808D showing the movement of the cytoplasmic shell from the most upward-inward conformation (cyan) to the most downward-outward conformation (red) from the top view.
